# NOX4-derived ROS are neuroprotective by balancing intracellular calcium stores

**DOI:** 10.1007/s00018-023-04758-z

**Published:** 2023-04-21

**Authors:** Lukas Gola, Laura Bierhansl, Júlia Csatári, Christina B. Schroeter, Lisanne Korn, Venu Narayanan, Manuela Cerina, Sara Abdolahi, Anna Speicher, Alexander M. Hermann, Simone König, Albena T. Dinkova-Kostova, Tawfeeq Shekh-Ahmad, Sven G. Meuth, Heinz Wiendl, Ali Gorji, Matthias Pawlowski, Stjepana Kovac

**Affiliations:** 1 Department of Neurology with Institute of Translational Neurology, University Hospital Münster, 48149 Münster, Germany; 2 Department of Neurology, Medical Faculty, Heinrich Heine University Düsseldorf, 40225 Düsseldorf, Germany; 3 Shefa Neuroscience Research Center, Khatam Alanbia Hospital, Tehran, Iran; 4 Core Unit Proteomics, Interdisciplinary Center for Clinical Research, Medical Faculty, University of Münster, 48149 Münster, Germany; 5 Division of Cellular Medicine, School of Medicine, University of Dundee, Dundee, DD1 9SY UK; 6 Institute for Drug Research, The School of Pharmacy, Faculty of Medicine, The Hebrew University of Jerusalem, 91120 Jerusalem, Israel; 7 Epilepsy Research Center, Westfälische Wilhelms-Universität Münster, 48149 Münster, Germany

**Keywords:** Hyperexcitability, Reactive oxygen species (ROS), NADPH oxidase-4 (NOX4), Mitochondria, Calcium signaling, Neurodegeneration

## Abstract

Hyperexcitability is associated with neuronal dysfunction, cellular death, and consequently neurodegeneration. Redox disbalance can contribute to hyperexcitation and increased reactive oxygen species (ROS) levels are observed in various neurological diseases. NOX4 is an NADPH oxidase known to produce ROS and might have a regulating function during oxidative stress. We, therefore, aimed to determine the role of NOX4 on neuronal firing, hyperexcitability, and hyperexcitability-induced changes in neural network function. Using a multidimensional approach of an in vivo model of hyperexcitability, proteomic analysis, and cellular function analysis of ROS, mitochondrial integrity, and calcium levels, we demonstrate that NOX4 is neuroprotective by regulating ROS and calcium homeostasis and thereby preventing hyperexcitability and consequently neuronal death. These results implicate NOX4 as a potential redox regulator that is beneficial in hyperexcitability and thereby might have an important role in neurodegeneration.

**Supplementary Information:**

The online version contains supplementary material available at 10.1007/s00018-023-04758-z.

## Introduction

The hallmark of neuronal tissue is its excitable nature, which is determined by neuronal firing. Unsurprisingly, this neuronal excitability can become overwhelming resulting in hyperexcitability, which is an underlying disease mechanism in many brain pathologies such as epilepsy, stroke, neuropathic pain and Alzheimer’s disease [1–3]. Hyperexcitability has been recognised decades before as a hallmark of epilepsy but the recent investigation also demonstrated hyperexcitability in other neurological diseases such as demyelinating brain diseases like multiple sclerosis, as well as neurodegenerative diseases [4–6]***.*** Hyperexcitability itself has been found to contribute to neurodegeneration and neuronal cell death [7]. The cellular mechanisms leading to such neurodegeneration and cell death unequivocally invoke metabolic compromise through bioenergetic failure and excess reactive oxygen species (ROS) which are conserved mechanisms of cell death pathways [8]. Excess mitochondrial ROS are the leading cause of cell damage in many brain diseases where neurodegeneration is a prominent feature, such as in dementia [9]. Additionally, ROS have been linked to hyperexcitability as mice lacking superoxide dismutase (SOD), a ROS detoxifying enzyme, exhibit spontaneous seizures, but the sources of ROS regulating hyperexcitability remain elusive [10].

Among these, the NADPH oxidase (NOX) family should be mentioned since it is the only known enzyme family that is specifically dedicated to ROS production [11]. Initially discovered in phagocytic cells, NOX-derived ROS have been shown to mediate pathogen defense [12]. The development of more sensitive assays led to the detection of NOX-derived ROS in non-phagocytic cells [13] and supported the notion that ROS production through NOX is not only detrimental to the cell but holds also important functions in cell signaling [14]. As such, it could be an ideal candidate to link metabolic homeostatic changes with neuronal excitability within neural tissue.

There are seven isoforms of NOX reported in most mammals [15]. Of those, NOX1, 2, 3 and 4 isoforms are mainly expressed in the human brain [16, 17]. The role of NOX2 has been investigated in depth in neuronal tissue, where it has an important role in homeostatic functions such as LTP, but also acts as a key ROS producer leading to cell death during hyperexcitability and excitotoxic cellular stress [18, 19]. NOX1 shares a 60% sequence identity with NOX2 and has been implicated in pain aggravation [20] and processing after inflammation [21]. Similar to NOX2, NOX4-derived ROS have also been implicated in brain diseases, where ROS generation through NOX4 mediates neuronal damage in brain ischemia through NOX4-derived ROS from both endothelial cells and neurons [22, 23]. However, also beneficial and conflicting results have been published in other cell types such as myocardiocytes [24], retina [25] or endothelial cells [26], whereas NOX4 has a pro-survival role.

These conflicting results and difficulties in examining the role of NOX may be a consequence of the fact that NOX4 in contrast to the other subforms is constitutively active [27, 28] and as such bears a greater potential of regulation. Hence, it is surprising that there are no investigations into the role of NOX4-derived ROS in homeostatic/basal brain function. In ischemia or hypoxia models NOX4 has been implicated to mediate the ischemic ROS excess [23]. In contrast to ischemia, hyperexcitability represents a different type of cellular stress, whereas calcium homeostasis, mitochondrial function and ROS all have been shown to play a major role in hyperexcitability-induced neuronal damage [7]. More recently, NOX4 is expressed in mitochondria and mitochondria-endoplasmatic contact sites rendering it an ideal candidate to link calcium homeostasis and metabolic processes to cell fate and hyperexcitability [24]. Furthermore, an ATP-binding motif within NOX4 has been shown to function as a mitochondrial energetic sensor which inhibits NOX4-derived ROS production and thereby promotes cellular pro-survival pathways in renal carcinoma cells [29]. Such properties render NOX4 ideal to link mitochondrial bioenergetic failure to cell death which has been implicated in hyperexcitability-induced neuronal cell death [7].

We, therefore, aimed to determine the role of NOX4 on neuronal firing, hyperexcitability, and hyperexcitability-induced neuronal damage.

## Results

### NOX4 is expressed in human epileptic tissue, in murine glio-neuronal and in iPSC-derived neuronal tissue with a cluster at ER and mitochondria contact sites

**Fig. 1:**
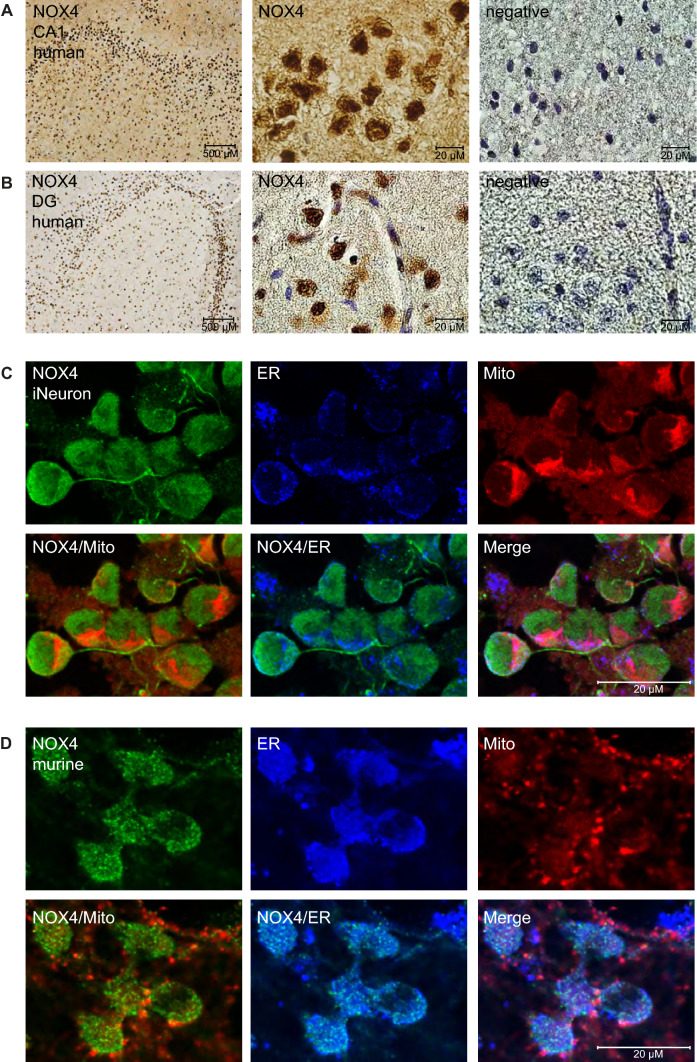
Immunohistochemical analysis of NOX4 localization. Immunohistochemistry of ex vivo human epileptic tissue, and immunoflourescence in vitro human iPSC-derived neuronal cultured cells and in vitro murine glio-neuronal cultured cells. **A**, **B** Immunohistochemistry revealed a strong NOX4 Expression in CA1 (**A**) and DG (**B**) of hippocampal epilepsy surgery tissues. **C** Immunofluorescence of expression/distribution of NOX4 in iNeurons costained with Mitotracker Deep Red to visualize mitochondria (Mito) and Calnexin to visualize endoplasmatic reticulum (ER). **D** NOX4 expression in murine glio-neuronal cells at the ER site in close proximity to mitochondria demonstrated by mitochondrial staining (Mito) with Mitotracker Deep Red and endoplasmatic reticulum (ER) staining with ER-Tracker Blue White

NOX4 is known to be expressed in neurons, however knowledge about the distribution within the brain and the expression in human tissue is rather limited. Here we explored the expression of NOX4 in epileptic tissue. Therefore, we analyzed the NOX4 expression in human hippocampal brain sections obtained from epilepsy surgery. We observed NOX4 expression within the hippocampus, with a pronounced regional expression of NOX4 in cornu ammonis (CA1) and dentate gyrus (DG) in neurons, glia cells and endothelial cells (Fig. 1a, b). To perform more detailed analyses of the subcellular localization of NOX4 we performed expression analysis in murine and human (iPSC-derived) neuronal cultures, rendering them suitable as model systems to further study the role of NOX4 in hyperexcitability and neuronal activity. NOX4 expression in human-induced neurons (iNeurons) was seen pronounced in the cytoplasm (Fig. 1c), but was also expressed within the nucleus. To specifically address subcellular NOX4 distribution within neurons we performed co-stainings of NOX4 with mitotracker (Mito) and endoplasmatic reticulum tracker (ER) in murine and human neurons, which showed NOX4 localization at the ER site in close vicinity to mitochondria (Fig. 1c, d), confirming previous studies which were performed in other tissues [24].

### NOX4 knockout (NOX4^−/−^) mice show enhanced neuronal activity during baseline and in remyelination in the cuprizone model of de- and remyelination

**Fig. 2:**
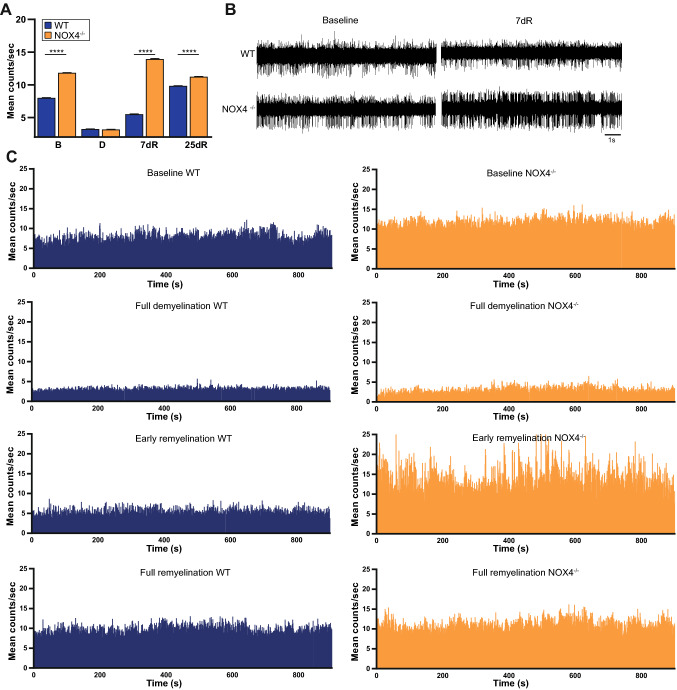
Hyperexcitability in vivo. The effect on neuronal firing in the cuprizone model in NOX4^−/−^ mice and WT littermates in the hippocampus was analyzed at baseline (B; WT, n = 60; NOX4^−/−^, n = 54), full demyelinaton (D; WT, n = 75; NOX4^−/−^, n = 41), early (7dR; WT, n = 43; NOX4^−/−^, n = 30) and full remyelination (25dR; WT, n = 44; NOX4^−/−^, n = 30). **A** Bar graphs summarizing single unit analysis. **B** Example of spike activity before and after cuprizone treatment. **c** Graphs showing neuronal activity evoked in CA1 at baseline, after cuprizone treatment and reintroducing to normal food. Data information: Data are mean ± SEM; one-way ANOVA; n ≙ number of neurons out of 7–10 mice per genotype and time point; ****p < 0.0001

Neuronal excitability is a fundamental capability of neurons to sustain brain function and synaptic transmission. To evaluate the effect of NOX4 within a dysfunctional network as seen in neuroinflammation or during epileptic activity, we choose a model of hyperexcitability in vivo that renders the dysfunctionality within the neurological disease. We have recently described that remyelination within the cuprizone model is associated with a period of hyperexcitability in the cortex [30]. The cuprizone model induces de- and remyelination with secondary changes in the cortical organisation [30], which therefore is suitable to evaluate the effect of NOX4 on neuronal activity and hyperexcitability. In vivo electrophysiological recordings and single unit analysis were performed in the CA1 region of WT and NOX4^−/−^ mice. This showed that neuronal firing rate was increased at pre-diet condition (Baseline; B) in NOX4^−/−^ mice when compared to WT littermates (Fig. 2a–c). The differences in neuronal firing rates between WT and NOX4^−/−^ animals were even more pronounced in early remyelination (7dR; Fig. 2c) with higher neuronal firing rates in NOX4^−/−^ mice. This difference was also observed in full remyelination (i.e. 25 days after reintroduction of normal diet), although with a smaller effect size (Fig. 2c).

**Fig. 3:**
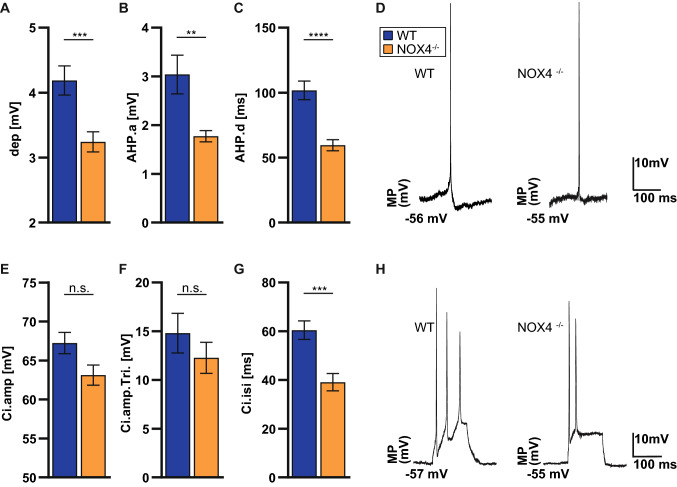
Intracellular recordings ex vivo. **A**–**C** Histograms showing, the depolarization (dep), amplitude of after-hyperpolarization (AHP.a) and its duration (AHP.d) during intracellular recordings (WT, n = 96; NOX4^−/−^, n = 103). **D** Exemplary traces of intracellular recordings. **E**–**G** Bar charts showing the analysis of interspike intervals between first and second action potential (AP) triggered by current injections (Ci). Maximum amplitude of the first AP (Ci.amp; WT, n = 21; NOX4^−/−^, n = 41), after triggering (Ci.amp.Tri.; WT, n = 57; NOX4^−/−^, n = 97), and interspike interval between the first and the second AP (Ci.isi; WT, n = 31; NOX4^−/−^, n = 51). **H** Exemplary traces of typical discharge patterns of CA1 neurons after triggered by Ci. Data information: Data are mean ± SEM; Student’s t-test; n ≙ AP out of 12 ROI (region of interest) of 6 WT mice and 14 ROI of 5 NOX4^−/−^ mice; **p < 0.01, ***p < 0.001, ****p < 0.0001

Higher in vivo firing rates both at baseline and during early remyelination in NOX4^−/−^ mice could be explained by differences in action potential thresholds. To investigate this in more depth, we performed intracellular recordings in neurons in CA1 regions from NOX4^−/−^ and WT littermate mice. In keeping with the observed in vivo hyperexcitability in NOX4^−/−^ mice, intracellular recordings showed, with regard to the membrane potential, a lower action potential depolarization threshold (dep; Fig. 3a), a smaller after-hyperpolarization threshold (AHP.a; Fig. 3b) and shorter after-hyperpolarization duration time in NOX4^−/−^ mice (AHP.d; Fig. 3c), indicating the potential effect of NOX4 on the regulation of neuronal membrane potential and neuronal excitability. Additional intracellular recordings were made by using current injections (Ci) to trigger action potentials in CA1 neurons (Fig. 3e, h). NOX4^−/−^ mice showed a shorter intervall between the first and the second action potential (Ci.isi; Fig. 3g). These membrane potential fluctuations together with the fast generation of Ci-triggered action potentials suggest a higher propensity to generate action potentials in NOX4^−/−^ neurons compared to controls.

### The cellular ROS production is enhanced in ex vivo brain slices of NOX4^−/−^ mice during de- and remyelination in the cuprizone model

**Fig. 4:**
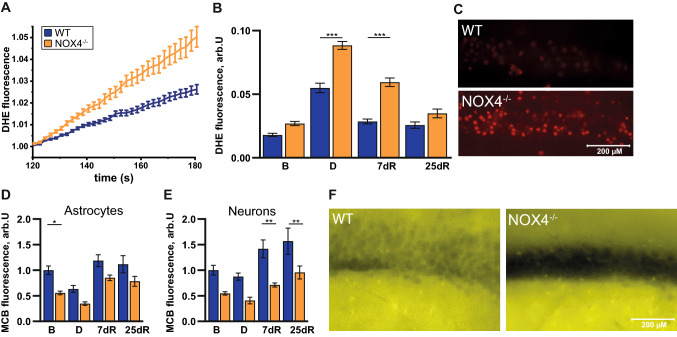
Analyses of redox homeostasis in the cuprizone model in vivo. **A** Graphs showing traces of a representative experiment of DHE-stained murine slices at full demyelination. **B** Bar charts summarizing levels of ROS in hippocampal CA1 slices at baseline (B; WT, n = 60; NOX4^−/−^, n = 64), full demyelination (D; WT, n = 58; NOX4^−/−^, n = 57), early remyelination (7dR; WT, n = 44; NOX4^−/−^, n = 52) and full remyelination (25dR; WT, n = 44; NOX4^−/−^, n = 48). **C** Exemplary images of DHE-stained CA1 hippocampal slices after full demyelination in WT and NOX4^−/−^ mice. **D**, **E** Histogramms summarizing glutathione responses to cuprizone treatment at all 4 time points in CA1 of hippocampal slices in astrocytes (B: WT, n = 34; NOX4^−/−^, n = 33; D: WT, n = 24; NOX4^−/−^, n = 30; 7dR: WT, n = 29; NOX4^−/−^, n = 29; 25dR: WT, n = 33; NOX4^−/−^, n = 25) and in neurons (B: WT, n = 36; NOX4^−/−^, n = 35; D: WT, n = 35; NOX4^−/−^, n = 33; 7dR: WT, n = 30; NOX4^−/−^, n = 33; 25dR: WT, n = 35; NOX4^−/−^, n = 31). **F** Exemplary images representing MCB-stained CA1 hippocampal slices at full demyelination in WT and NOX4^−/−^ mice. Data information: Data are mean ± SEM; one-way ANOVA; n ≙ glio-neuronal cells out of 4 mice per genotype and time point, each with 2 brain slices; *p < 0.05, **p < 0.01, ***p < 0.001

Excess ROS, such as those induced by a lack of antioxidants, lead to hyperexcitability [10]. Thus, an increase in neuronal firing in NOX4^−/−^ mice compared to WT is surprising, since NOX4 is known to generate ROS. We, therefore, assessed ex vivo ROS production in NOX4^−/−^ mice and littermate controls. Unexpectedly, we found higher neuronal ROS production rates in the CA1 region of mice where NOX4 is inactivated as a ROS source (NOX4^−/−^; Fig. 4a–c) in keeping with higher neuronal firing rates in NOX4^−/−^ mice (Fig. 2). Temporal analysis of ROS production during de- and remyelination in the cuprizone model showed that the increase in the rate of ROS production in NOX4^−/−^ mice was particularly pronounced during both demyelination and early remyelination (Fig. 4b). This is rather surprising, but may indicate that the ROS derived from NOX4 may negatively regulate neuronal activity, which leads to hyperexcitability during remyelination in NOX4^−/−^ mice, whereas ROS levels during remyelination in WT animals are not affected. However, demyelination led to excessive ROS production within this model in WT and at an even higher extent in NOX4^−/−^ mice, suggesting a neuroinflammatory-induced overload of ROS production, which leads to dysfunctional neuronal activity and thereby reduced neuronal excitability (neuronal firing rates) independent of NOX4 ROS signaling because of the oversaturated ROS levels. In accordance with the changes in ROS production, we further investigated the levels of glutathione (GSH), which is a major ROS scavenger in the brain [31, 32]. GSH is supplied to neurons mainly via astrocytes and correlates negatively with ROS levels, since ROS is reduced by the GSH/GSSG (Glutathione disulfide) system. In line with the increased ROS levels, we observed a reduction in GSH in neurons but also in astrocytes in NOX4^−/−^ neurons during the different stages of the cuprizone model (Fig. 4d–f). This decrease varied with the most prominent reduction in glutathione levels seen in neurons in CA1 during early (7d) and full (25d) remyelination (Fig. 4e).

### Proteomic analyses reveal alterations of calcium signaling, metabolic processes and signaling in NOX4^−/−^ glio-neuronal cultures

**Fig. 5:**
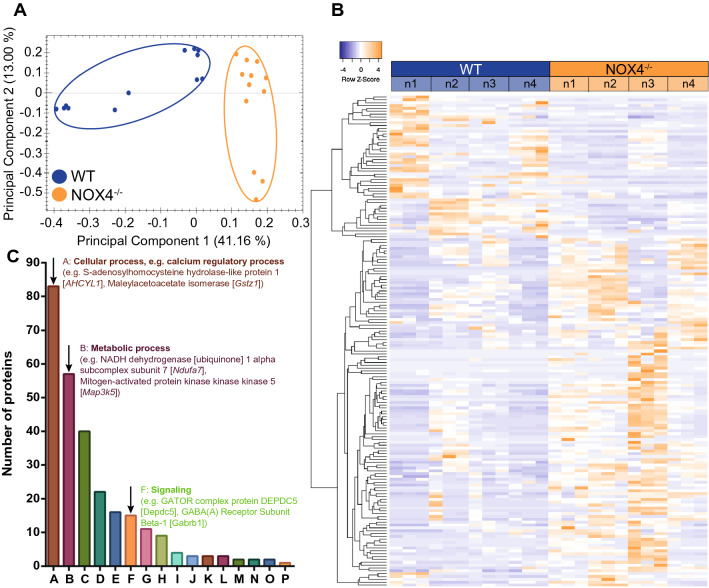
Mass spectrometry-based proteomic comparative analysis of NOX4^−/−^ neurons. Label free quantification of high-resolution mass spectrometry data was performed on four biological replicates (n = 4) of both WT and NOX4^−/−^ mice. Each biological replicate was measured in three technical replicates. **A** Principal component analysis of all technical and biological replicates shows clustering of the two experimental groups with a variance of 41.16% in principal component 1 and 13.00% in principal component 2. **B** Heatmap of shortlisted proteins. **C** Gene ontology analysis using the Panther classification system of significantly regulated biological processes: cellular process (A), metabolic process (B), biological regulation (C), response to stimulus (D), localization (E), signaling (F), multicellular organismal process (G), developmental process (H), locomotion (I), biological adhesion (J), immune system process (K), interspecies interaction between organisms (L), biological phase (M), reproduction (N), reproductive process (O) and growth (P). Data information: One-way ANOVA, p $\le$ 0.05, fold change $\ge$ 2

Due to the observation of increased ROS levels and enhanced excitability in NOX4^−/−^ mice, we aimed to further characterize the function of NOX4 in neuronal redox homeostasis and signaling and we, therefore, performed mass spectrometry-based proteomic analyses in WT and NOX4^−/−^ cultures (Fig. 5). Using this unbiased approach, we clear hierarchical clustering of proteins in NOX4^−/−^ and WT glio-neuronal cultures (Fig. 5a). Correlation heatmap analysis illustrated a difference in protein expression signatures in WT vs. NOX4^−/−^ cells (Fig. 5b). Further analysis of significantly regulated gene ontology (GO) terms indicated shifts in basic cellular function such as calcium homeostasis, metabolic processes but also signaling, all of which are important in excitability and hyperexcitability (Fig. 5c). To further dissect these processes which contribute to hyperexcitability in NOX4 deficiency, we chose to study calcium and ROS homeostasis in accordance with metabolic changes using live cell imaging in glio-neuronal NOX4^−/−^ and WT cultures.

### ROS levels in NOX4^−/−^ glio-neuronal cultures are increased potentially due to impaired Nrf2 activity

**Fig. 6:**
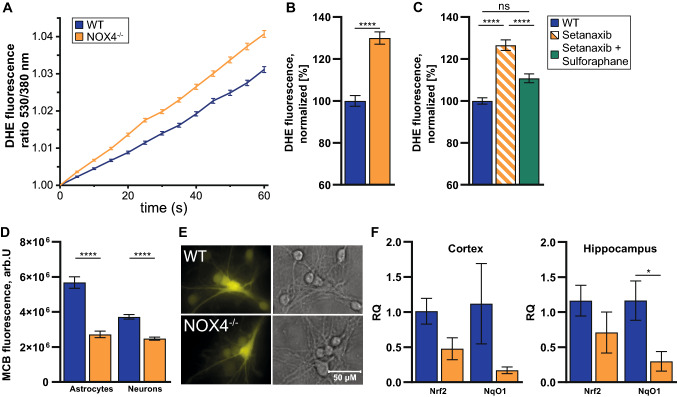
Impaired ROS homeostasis and mRNA expression of Nrf2 target gene changes in glio-neuronal cultures. **A** Representative experiment of DHE fluorescence over time in glio-neuronal cultures. **B** ROS levels in WT (n = 364) and NOX4^**−/−**^ (n = 274) glio-neuronal cultures. Fluorescence was analyzed after 120 s DHE application and is presented as fluorescence per second. **C** Normalized DHE fluorescence of NOX4-proficient glio-neuronal cells (n = 121), NOX4-inhibited glio-neuronal cells (Setanaxib; n = 155) and NOX4-inhibited and Nrf2-activated glio-neuronal cells (Setanaxib + Sulforaphane; n = 113). **D** Glutathione levels in glio-neuronal cultures incubated with MCB in astrocytes (NOX4^−/−^, n = 175; WT, n = 168) and in neurons (NOX4^−/−^, n = 401; WT, n = 472). **E** Exemplary images of MCB-stained cultures. **F** mRNA expression levels of Nrf2 (*Nfe2l2*) and its target gene *Nqo1* in cortex and hippocampus of WT (n = 5) and NOX4^**−/−**^ (n = 5) tissues of adult mice (8–12 weeks). All expression levels were normalized to gene expression in WT. Data information: Data are mean ± SEM; Student’s t-test; **B** n ≙ number of glio-neuronal cells out of 3 independent neuronal preparations, each with 2 CS; **C** n ≙ number of glio-neuronal cells out of 3 independent neuronal preparations with 7–8 CS per condition; **D** n ≙ number of glio-neuronal cells out of 3 independent neuronal preparations, each with 2 CS and 5 ROI per CS; **F** n ≙ number of mice; *p < 0.05, ****p < 0.0001

We first asked whether our key finding of higher rates of ROS production in NOX4^−/−^ neurons ex vivo (Fig. 4) can be replicated in vitro in NOX4^−/−^ glio-neuronal cultures. This would be a suitable model for 2D live cell imaging to provide further insight into calcium handling and metabolic processes, the two key processes revealed by the unbiased proteomic screen. Indeed, NOX4^−/−^ glio-neuronal cultures have higher ROS production rates (Fig. 6a, b), which corresponds to the lower glutathione levels in both neurons and astrocytes (Fig. 6d, e) in these cultures. The main regulator of glutathione biosynthesis and its maintenance in the reduced (GSH) state is transcription factor Nrf2 [33]. Analysis of the mRNA levels for *Nfe2l2*, the gene encoding Nrf2, and for its classical target gene, *Nqo1* revealed that they were lower in hippocampal tissue of NOX4^−/−^ mice when compared to control tissue (Fig. 6f). Furthermore, treatment with the pharmacological NOX4 inhibitor Setanaxib (GKT137831) also increased ROS production, which was partially restored upon Nrf2 induction with sulforaphane (Fig. 6c). In conclusion, these results suggest that ROS production in NOX4^−/−^ glio-neuronal cultures is partially explained by Nrf2 pathway hypofunction.

### A lack of NOX4-derived ROS leads to pronounced cell death in the low magnesium model of hyperexcitability which is promoted through excess oscillatory intracellular calcium

**Fig. 7:**
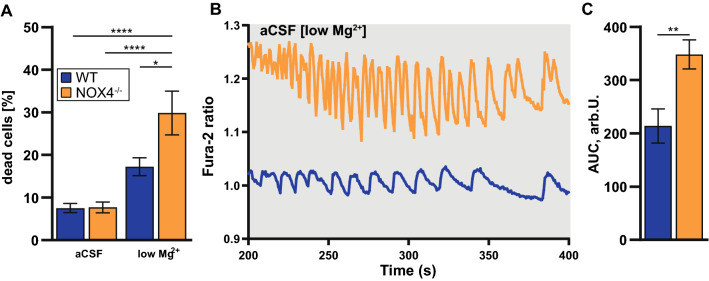
Analyses of cell death and calcium oscillation in NOX4^−/−^ glio-neuronal cells in hyperexcitability. **A** Analysis of co-staining of glio-neuronal cells (n = 25) with propium iodide and Hoechst 33342 to evaluate cell death. Hyperexcitability was induced by a reduction of magnesium in aCSF. **B** Representative graphs of hyperexcitability-induced calcium oscillations. Calcium signaling in glio-neuronal co-cultures were measured using the Ca^2+^-dye Fura-2 AM. **C** Bar graphs showing area under the curve of low magnesium triggered calcium oscillations in WT (n = 30) and NOX4^−/−^ cells (n = 30). Data information: Data are mean ± SEM; Student’s t-test; **A** n ≙ number of ROI out of 3 independent neuronal preparations each with 1–2 CS; **B** n ≙ number of glio-neuronal cells out of 2 independent neuronal preparations, each with 2 CS; *p < 0.05, **p < 0.01, ****p < 0.0001

We next asked whether NOX4 deficiency is beneficial with regard to neuronal survival during hyperexcitability. We, therefore, studied neuronal cell death in the low magnesium model of hyperexcitability [34, 35]. In the baseline condition, NOX4^−/−^ did not affect the cell viability, but NOX4^−/−^ glio-neuronal cultures were more susceptible to low magnesium-induced neuronal cell death (Fig. 7a), suggesting a protective role of NOX4 in hyperexcitability. Previously it was shown that hyperexcitability can induce neuronal death due to increased intracellular calcium oscillation and subsequent calcium overload leading to mitochondrial dysfunction [7]. We, therefore, observed the oscillatory calcium signal in both WT and NOX4^−/−^ glio-neuronal cultures (Fig. 7b). There was a difference in the calcium burden between the different cultures with higher areas under the curve (Fig. 7c) and thus higher overall hyperexcitability-induced calcium burden seen in glio-neuronal cultures from NOX4^−/−^ animals when compared to controls. These increased calcium oscillations may have contributed to higher neuronal cell death in NOX4^−/−^ cultures subjected to low magnesium-induced hyperexcitability.

### Mitochondrial calcium stores overload and mitochondrial membrane potential depolarization lead to a decrease in bioenergetic reserve and contribute to neuronal cell death

**Fig. 8:**
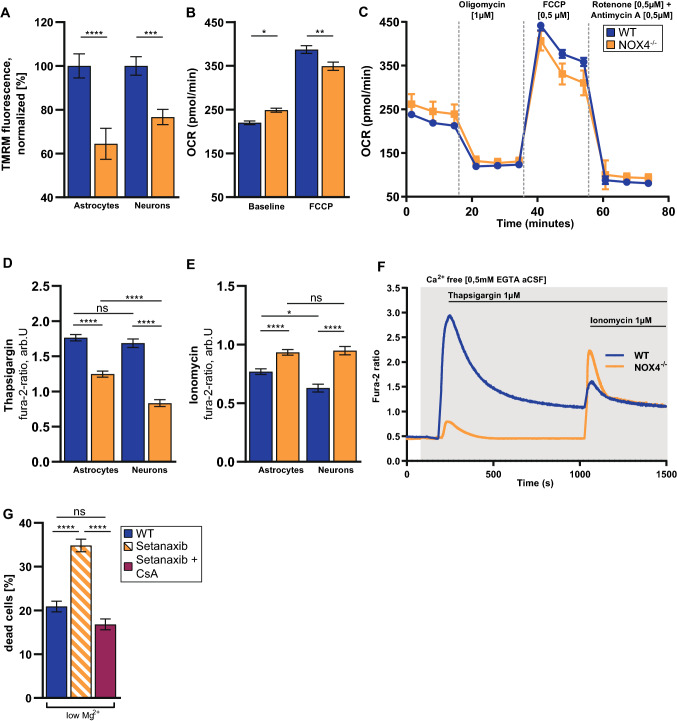
NOX4^−/−^ depolarized mitochondrial membrane potential and compromised the bioenergetic reserve. **A** Bar charts showing basal mitochondrial membrane potential measured with TMRM in astrocytes (WT, n = 75; NOX4^−/−^, n = 58) and neurons (WT, n = 144; NOX4^−/−^, n = 89). **B** OCR quantification under basal conditions and under mitochondrial stress (n = 9). **C** Graph showing OCR over time in glio-neuronal cells. **D**, **E** Bar charts summarizing the effect of thapsigargin and subsequently ionomycin application on intracellular calcium levels in astrocytes (WT, n = 377; NOX4^−/−^, n = 423) and neurons (WT, n = 153; NOX4^−/−^, n = 189). The calcium chelator EGTA was added to aCSF to guarantee a calcium free environment. **F** Representative traces showing the effect of thapsigargin and ionomycin on intracellular calcium levels in WT and NOX4^−/−^ murine neurons. **G** Bar charts showing the effect of low Mg^2+^-induced cell death in NOX4-proficient glio-neuronal cells (n = 30), NOX4-inhibited glio-neuronal cells (n = 30) and NOX4-inhibited and mPTP blocked glio-neuronal cells (n = 30). Data information: Data are mean ± SEM; Student’s t-test; **A** n ≙ number of glio-neuronal cells out of 3 independent neuronal preparations, each with 2CS and 5 ROI per CS; **B**, **C** n ≙ number of wells seeded with glio-neuronal cells out of 3 independent neuronal preparations; **D**, **E** n ≙ number of glio-neuronal cells out of 5 independent neuronal preparations; 13 CS with WT cells and 12 CS with NOX4^−/−^ cells; **G** n ≙ number of ROI out of 3 independent neuronal preparations each with 2 CS; *p < 0.05, **p < 0.01, ***p < 0.001, ****p < 0.0001

Neurons critically depend on a mitochondrial function to establish membrane excitability [36]. Mitochondria are the hub of calcium signaling and main energy producers particularly within neurons [37, 38]. We thus hypothesized that mitochondrial function will be affected in NOX4^−/−^ when compared to WT neurons. In line with previous studies [24], we found that NOX4 is expressed in the vicinity to mitochondria (Fig. 1c, d). Morphological analysis of the mitochondria showed a reduction in mitochondria size and reduced branch length, indicating a fragmentation of the mitochondria in NOX4^−/−^ cultures (Appendix Fig. S1a–d). Mitochondrial fragmentation is associated with dysfunctional mitochondrial activity and alteration in energy homeostasis [39]. In accordance we found that the mitochondrial membrane potential as measured with tetramethylrhodamine (TMRM) is depolarized (Fig. 8a) and that baseline respiration is increased in NOX4^−/−^ cells with reduced maximal respiration capacity when compared to controls (Fig. 8b, c), indicating energy disbalance with exhausted mitochondrial respiration under NOX4 deficiency.

Next, we analyzed intracellular calcium dynamics in astrocytes and neurons under NOX4^−/−^ condition. Here we observed a decrease in the calcium release from the ER (Fig. 8d, stimulation with thapsigargin) with a compensatory increase in calcium release from the mitochondria in NOX4^−/−^ neurons when compared to WT controls (Fig. 8e, stimulation with ionomycin), suggesting that NOX4 may be involved in the intracellular calcium handling in keeping with its strategic localization at mitochondria ER contact sites (Fig. 1c, d). However, unless no focus of this study further analyses are needed to differentiate the calcium dynamics between these organelles. Together with mitochondrial depolarization, mitochondrial calcium overload during hyperexcitability in NOX4^−/−^ neurons constitutes a strong trigger to mitochondrial permeability transition pore (mPTP) opening leading to cell death. And indeed pharmacological blockage of the mPTP opening by Cyclosporin A (CsA) can abolish the effects of the increased cell death under NOX4 inhibition (Fig. 8g).

## Discussion

Hyperexcitability is a hallmark of various neurological diseases and is associated with neuronal dysfunction, cellular death, and consequently neurodegeneration. Therefore, understanding the molecular mechanism and identification of potential regulators can lead to future strategies for the prevention and therapy of neurodegenerative processes.

The role of ROS within the pathomechanism of neurodegeneration has been examined for decades [40], however, the origin of the species and if they are rather beneficial or detrimental is still the subject of many discussions. The NADPH oxidases (NOX), a group of multisubunit enzymes, are in focus because of their unique function as a key producer of ROS in many cells. Within the brain, different subtypes of NOX have been detected. NOX2 has been investigated extensively, whereas the knowledge of the function of NOX4 in CNS is rather limited, even if expression analysis in disease models suggests an equivalent increase of NOX2 and NOX4 expression within pathological stress conditions [41]. In addition, NOX4 expression is also upregulated in patients with Alzheimer’s disease or frontotemporal dementia [42], questioning the role of NOX4 in neurodegeneration.

Here, we demonstrated that NOX4 is expressed in human brain tissue, and neuronal NOX4 expression is closely located at the interface of mitochondria and endoplasmatic reticulum (ER), indicating that NOX4 is a potential regulator between those cellular components. Calcium influx is essential for neuronal excitability and recently it was demonstrated that stress-induced upregulation of NOX4 at the ER-mitochondria contact sites is a pro-survival mechanism that inhibits calcium transfer in cardiomyocytes, leading to reduced calcium flux and mPT- (mitochondrial permeability transition) dependent cardiac cell death [24]. In our model NOX4 deficiency led to increased calcium oscillations in neurons, and dysfunctional mitochondria, indicated by changes in morphology, depolarization, and exhausted respiration. These effects result in an accelerated susceptibility to hyperexcitability and consecutive calcium overload associated neuronal death in NOX4-deficient cells, indicating a protective role of NOX4 in neurons in hyperexcitability.

Since NOX4 is an enzyme that produces ROS, we further analyzed the ROS levels in the state of hyperexcitability in vivo and in vitro. Here, we demonstrated that the deficiency of NOX4 leads to an increase in the neuronal activity, implicating a hyperexcitable state during de- and remyelination in vivo, which was associated with increased ROS levels. However, this is rather counterintuitive since NOX4 is known to generate ROS, and NOX4 deficiency was shown to reduce ROS levels in other disease models before [22, 23, 43]. Additionally, NOX4^−/−^ mice did not show changes in the expression levels of NOX2 within the brain (data not shown), an observation known from other tissues before [44, 45]. More interestingly, NOX4 expression is elevated during hypoxia and brain ischemia model [46], after intracerebral hemorrhage [47] and traumatic brain injury [43], all stress conditions associated with ROS excess and detrimental neuronal damage. Thereby we questioned if the elevated NOX4 levels are involved in this neuronal damage or if this is a compensatory mechanism since NOX4 might induce an antioxidative response. In contrast, the ROS excess during hyperexcitability is rather moderate, potentially explaining the different effects on ROS levels by the inhibition of NOX4. In physiological conditions, NOX4 might therefore act as a sensor and effector that balance the intracellular ROS homeostasis. Within the brain, the sensitivity of CNS cells to ROS is likely a key factor in the signaling versus cell death decision [48]. Thereby, redox-regulated transcription factors such as *Nrf2* and/or NOX4 might be involved in stress response, rather than being involved in CNS-specific pathways. And indeed, it is known that NOX4 can function as a mitochondrial energetic sensor that promotes cellular pro-survival pathways in renal carcinoma cells [49]. Furthermore, during neurogenesis, NOX4 is essential for hippocampal development and efficient neuronal differentiation of neuronal stem cells [50] and NOX4 can promote neuronal stem/precursor cell proliferation after injury [51] highlighting NOX4 as a pro-survival regulating factor.

In our model, NOX4 seems to have a redox sensing function, and the moderate ROS disbalance in the hyperexcitable state can induce the NOX4-dependent antioxidative response. The increase in ROS levels in NOX4^−/−^ neurons might be an effect of the missing redox regulating properties of NOX4. We hypothesized that in the absence of NOX4, the activity of transcription factor Nrf2, which is activated by the NOX4 product, hydrogen peroxide [52], is low. Indeed, the deletion of NOX4^−/−^ was accompanied by a reduction of the expression of *Nqo1*, a well-known Nrf2-target. Nrf2 is known to downregulate NOX4 expression, and thereby potentially modulate the redox balance [53–55]. Additionally, it has been shown that NOX4 overexpression leads to Nrf2 activation and induction of its target genes [56], whereas cardiomyocyte-specific NOX4-deficient mice are characterized by cardiac oxidative stress and absence of exercise-mediated Nrf2 activation [57]. Together with our observation that the increase in ROS levels in NOX4^−/−^ neurons was accompanied by a reduction in GSH production, these findings indicate that NOX4 is not solely a ROS producer, but is also involved in the production/reduction of glutathione, the most important endogenous ROS-scavenging small molecule.

Our results indicated that NOX4 has important protective effects within stress situation associated with hyperexcitability as neuroinflammation or neurodegeneration and these knowledge gives a new insight into the role of NOX4 in the low-stress model, pointing out that further therapies targeting NOX4 should be taken with caution. This together reflects that NOX4 might have a double-faced role within the CNS, depending on the pathological circumstances, whereas NOX4 might be beneficial in moderate stress levels by enhancement of the Nrf2-mediated antioxidant response, but in a situation of ROS oversaturation, NOX4 becomes dysfunctional or part of the cell death machinery, which leads to massive ROS production and consequently cell death. Therefore, modulating NOX4 function to rebalance the ROS levels to a physiological level, might be a future therapeutic strategy.

## Methods

### Murine neuronal cell cultures

Hippocampal glio-neuronal cell cultures were obtained from postnatal (P0–P2) C57BL6J (WT) mice and NOX4^−/−^ mice. Mice brains were quickly removed, and hippocampal tissue was isolated and transferred into ice-cold HBSS (Gibco). The tissue was treated with 5 ml Trypsin–EDTA (0.25%; Sigma-Aldrich) for 10 min at 37 °C to facilitate cell dissociation. Residual trypsin was removed and the tissue was triturated with fire-polished Pasteur pipettes, plated on poly-d-lysine (PDL, Sigma-Aldrich) pre-coated coverslips (CS) and cultured in neurobasal medium (Gibco) supplemented with B-27 (Gibco), 0.5 mM GlutaMAX (Gibco) and 1% penicillin/streptomycin (Sigma-Aldrich) at a density of 150,000 cells. Cultures were kept at 37 °C in a humidified atmosphere containing 5% CO_2_ for 12 to 20 days before experiments.

### Human iPSC-derived neuronal cultures

HiPSC-derived small molecule neural precursor cells (smNPCs) containing a doxycycline-inducible forward programming cassette for forced overexpression of NGN2 were cultured on Matrigel (354248, Corning)-coated wells in smNPC media (1:1 DMEM/F12, neurobasal media, 1% l-Glutamine, 1% N2-Supplement, 1% B27-supplement, 1% penicillin/streptomycin, 200 µM ascorbic acid, 0.5 µM SAG, 3 µM CHIR 99021) with media changes every 2–3 days according to a protocol described elsewhere [58]. For experiments, 150,000 cells were seeded on 12 mm CS and neuronal differentiation was induced by applying neuron media (DMEM/F12, 2% B27-supplement, 1% N2-Supplement, 1% penicillin/streptomycin, 10 ng/ml BDNF, 10 ng/ml NT-3, 0.2 mM cAMP, 200 µM ascorbic acid, 1 µg/ml doxycycline) and switching to half media change at day 10 after induction. Immunocytochemistry was performed with iNeurons between days 28 and 30 after induction.

### Mass-spectrometry based proteomics

Glio-neuronal cells of NOX4^−/−^ and WT mice were isolated and cultivated on CS as described before. Mass-spectrometry based proteomics was performed by the IZKF Core Unit Proteomics (Interdisciplinary Center for Clinical Research, Medical Faculty, University of Münster). For both experimental conditions, four biological replicates were collected. Consecutively, in vitro murine glio-neuronal cell cultures (1 ml in PBS) were centrifuged (30 min, 30,000*g*, 4 °C). The pellet was lysed in 200 µl buffer (4 M urea, 50 mM Tris, 4% SDS, and 10 mM TCEP) on ice using ultrasonic treatment (Labsonic M, Sartorius). Following centrifugation (30 min, 30,000*g*, 4 °C) the protein concentration was determined (Pierce BCA Protein Assay Kit). For preparation for protein identification, samples were placed in Pall Nanosep^®^ 10 K Omega filter units (10 kDa cut-off) and urea buffer (8 M urea, 100 mM Tris Base) was added to 200 µl followed by centrifugation [15 min, 12,500*g*, room temperature (RT)]. Washing of the analyte was repeated with 100 µl urea buffer in the same manner. For reduction (45 min), 100 µl 50 mM DTT in urea buffer was added to the filter unit. The unit was centrifuged and the sample was rinsed with 100 µl urea buffer. For alkylation, 50 mM iodoacetamide in urea buffer was placed into the filter unit. Incubation proceeded in the dark for 30 min at RT. Following centrifugation and rinsing with 300 μl 50 mM NH_4_HCO_3_ containing 10% acetonitrile (ACN) in urea buffer twice, 0.01 µg/µl trypsin in 50 mM NH_4_HCO_3_ containing 10% ACN (200 µl) were added to the filter unit. Incubation proceeded at 37 °C overnight (O/N). Peptides were collected by rinsing the filter with 5% ACN/0.1% formic acid thrice and centrifugated. They were dried using a Speedvac (ThermoScientific) and redissolved in 5% ACN/0.1% formic acid at 500 ng/µl. Peptide solutions (4 µl) were analyzed with three technical replicates by reversed-phase chromatography coupled to mass spectrometry with Synapt G2 Si/M-Class nanoUPLC (Waters Corp.). Data were analyzed using Progenesis (Nonlinear Diagnostics/Waters Corp.) and the Uniprot *mus musculus* database. One missed cleavage was allowed, carbamidomethylation was set as fixed and methionine oxidation as variable modifications, respectively. A shortlist of the protein output was created by demanding protein assignment by at least two peptides, a fold value of at least 2 and ANOVA p ≤ 0.05. Heatmaps were generated using the heat mapper software tool [59]. Gene ontology analysis of significantly regulated biological processes was performed using the Panther classification system [60].

### Live cell imaging and staining procedures

Live cell images were captured with an epifluorescence inverted microscope equipped with a 40 × oil-immersion and 20 × fluorite objective. Imaging was performed between 12 and 20 days after Neuronal preparation and dyes were diluted in artificial cerebrospinal fluid (aCSF; 120 mM NaCl, 2.5 mM KCl, 1.25 mM NaH_2_PO_4_, 22 mM NaHCO_3_, 25 mM glucose, 2 mM CaCl_2_, 2 mM MgSO_4_). The live cell imaging setup provided excitation light by an LED lamp passing through a monochromator at 340, 380 or 530 nm (Cairn Research, Faversham, UK). Emitted fluorescence was reflected with a long-pass filter to a cooled CCD camera (Retiga; QImaging) and digitalized to 12-bit resolution.

For detection of intracellular ROS production levels, cells were stained with 16 µM dihydroethidium (DHE, D11347, Invitrogen) and images were taken at an interval of 5 s. To prevent an accumulation of oxidized products, cells were imaged instantly without preincubation. DHE was not washed out and present during the whole recording time. For murine cultures ratiometric DHE fluorescence was measured with excitation light at 380 and 530 nm. Murine brain slices stained with DHE were excited by a single wavelength at 530 nm to avoid photobleaching and phototoxicity. The rate of ROS production increased from the beginning of the recording and a period of 60 s was used to calculate fluorescence per second. To analyze glutathione, glio-neuronal cells were stained with 50 µM Monochlorobimane (MCB, M1381MP, Invitrogen) for 40 min. Cells were excited by illumination at 380 nm and emitted light was detected at 510/80 nm. Murine slices were incubated in a staining chamber provided with carbonated aCSF at RT. After washing with aCSF slices were transferred to the recording setup and fixed with a self-made harp to prevent floating.

To investigate the difference in the basal mitochondrial membrane potential between NOX4^−/−^ and WT cells were stained with 30 nM tetramethylrhodamine, methyl ester (TMRM, T668, Invitrogen) for 40 min. TMRM was not washed out and present during the whole imaging. CS were imaged with excitation at 530 nm and emission at 705/72 nm.

A Cell death assay was performed to analyze neurotoxicity in glio-neuronal cultures incubated with low-Mg^2+^ aCSF for 2 h at 37 °C and 5% CO_2_. Cells were stained with Propidium iodide (PI; 10 µM) which stains only dead cells in combination with Hoechst 33342 (4.5 µM) which stains all nuclei and a percentage of PI-positive cells in correlation to all nuclei was calculated.

To determine intracellular calcium changes glio-neuronal cells were loaded with 5 µM Fura-2 AM (F1221, Invitrogen) and 0.005% Pluronic (Sigma-Aldrich) to facilitate Fura-2 entry into the cell. After 40 min incubation time cells were washed in aCSF and images were taken. Fura-2 AM-loaded cells were excited at 340 nm and 380 nm and the fluorescent emission was gathered at < 515 nm acquired with a timing of 1 s.

To measure mitochondrial and ER calcium stores Fura-2 loaded glio-neuronal cells were treated with 1 µM thapsigargin followed by 1 µM ionomycin in calcium free media (aCSF supplemented with 0.5 mM EGTA).

To analyze the effects of pharmacological NOX4 inhibition glio-neuronal cells were incubated with 10 µM Setanaxib (GKT137831, medchemexpress) for 48 h. Setanaxib-treated glio-neuronal cells were incubated with 5 µM of the Nrf2 activator Sulforaphane (Sigma-Aldrich) for 48 h in order to explore Nrf2/NOX4 interaction in glio-neuronal cultures. For the cell death assay 1 µM CyclosporinA (CsA) was added together with the low Mg^2+^ solution to the Setanaxib-treated glio-neuronal cells 2 h prior to Imaging.

### Quantitative real-time PCR

To evaluate mRNA expression levels of *Nrf2* and its target gene *Nqo1*, the Hippocampus and Cortex of mice between 8 and 12 weeks old were isolated and collected in RNAlater (Invitrogen). RNA extraction was performed using TRIzol (Invitrogen) and 500 ng RNA was synthesized to cDNA using the Maxima Reverse Transcriptase (Thermo Fisher).

For each quantitative real-time PCR (qPCR) reaction 40 ng cDNA was combined with Maxima Probe Rox qPCR Mastermix (Thermo Fisher) supplemented with adequate Taqman primers (Thermo Fisher) and 18S rRNA (Thermo Fisher) as an endogenous control using the StepOnePlus Real-Time PCR System (Applied Biosystems). Analysis was conducted with StepOne software v2.1. The following TaqMan Primers were used: Nrf2 (Mm00477784_m1) and Nqo1 (Mm01253561_m1).

Data were analyzed using the ∆∆CT method followed by relative quantification (2^−∆∆CT^).

### Seahorse

To assess mitochondrial respiration of glio-neuronal cells the oxygen consumption rate (OCR) was measured using a Seahorse XFp Extracellular Flux Analyzer (Agilent Technologies). One day before seeding, Seahorse XFp cell culture plates were coated with PDL (50 µg/ml) and one day before the measurement sensor cartridges and surrounding chambers were hydrated with calibrant buffer and incubated at 37 °C without CO_2_. Each Plate contains three wells of NOX4^−/−^ cells and WT cells. On the day before the neuron preparation cell culture plates were washed with PBS and 15,000 cells per well were seeded in neurobasal media for 12–18 days at 37 °C. Before the measurement cells have been washed and neurobasal media was replaced with aCSF supplemented with 10 mM sodium pyruvate. OCR was measured under basal conditions and in the presence of the mitochondrial stressors Oligomycin (1 µM), carbonyl cyanide-p-trifluoromethoxyphenylhydrazone (FCCP; 0.5 µM) and Rotenone (0.5 µM) combined with Antimycin A (0.5 µM). For data analysis Wave Software (Agilent Technologies) was used. After signal stabilization the cells were sequentially exposed to mitochondrial stressors.

### Human tissue preparation

Human tissues were collected from patients with medically intractable temporal lobe epilepsy during neurosurgical intervention*.* The hippocampal tissues were surgically resected en bloc and immediately immersed in cold phosphate-buffered saline (PBS; Gibco) with 10% penicillin–streptomycin (Gibco) and then transferred from the operating room to the laboratory within 5–10 min. The samples were fixed in 4% paraformaldehyde for 24–48 h, dehydrated, and embedded in paraffin. 4–5 µm sections were used for immunohistochemistry. DG and CA1 regions were examined under a light microscope (BX51, Olympus, Tokyo, Japan) and digitized with a high-resolution camera (10 × , 40 × and 100 × magnifications).

### Immunohistochemistry (IHC)

The sections of human tissues were mounted on glass slides after processing. Deparaffinization and rehydration were performed through a series of xylenes and decreasing concentrations of ethanol and washed three times with PBS (pH 7.4). The sections were subjected to antigen retrieval with Tris/EDTA buffer (pH 9.0) and heated on boiling water bath for 10–15 min. For blocking endogenous peroxidase activity, slides were submerged in 3% hydrogen peroxide diluted in methanol (20 min, RT) under dark conditions. After permeabilization with 0.1% Triton-X-100 (20 min, RT), sections were washed again and blocked with blocking buffer containing 0.5% BSA (Sigma-Aldrich), 5% NGS (Sigma-Aldrich) and 0.1% Triton X-100 in PBS for 1 h. Subsequently, sections were incubated with primary antibody against NOX4 (dilution 1:200; Rabbit monoclonal, Abcam, ab133303) O/N at 4 °C and rinsed with PBS three times. As secondary antibody HRP-conjugated goat anti-rabbit IgG (dilution 1:700; Abcam, ab97051) was applied for 60–90 min at RT. Finally, 3-3′-diaminobenzidine (0.05% DAB) solution (K3467, Dako Catalyzed Signal Amplification Kit) was used as a chromogen to visualize horseradish peroxidase activity. Hematoxylin was used for counterstaining. To validate the specificity of the immunostaining, negative control was processed by omitting primary antibodies.

### Immunocytochemistry (ICC)

ICC stainings were performed on iNeurons and murine glio-neuronal cells. CS containing iNeurons and murine glio-neuronal cells were fixed with 4% PFA, blocked with Roche blocking reagentor with 10% goat serum in PBS. For permeabilization PBS supplemented with 10% goat serum and 0.03% Triton X-100 (Sigma) was applied for 10 min. Samples were stained with primary antibody O/N at 4 °C and with the secondary antibody for 1 h at RT. For Mitotracker deep red FM (Mito) and ER-Tracker Blue-White DPX (ER) stainings, CS were incubated at 37 °C and 5% CO_2_ for 45 min before fixation. CS were mounted in Fluoromount-G™ Mounting Medium (00-4958-02, Invitrogen). Cells were analyzed using a confocal Leica SP8 microscope (Leica LAS X 3.5.6.21594 software).

### Cuprizone mice

For all in vivo cuprizone experiments, we used littermates by backcrossing NOX4^−/−^ mice with C57BL/6 WT mice. All WT littermates were proved to have NOX4 incorporated by genotyping and were not included in the experiments before reaching the third generation.

### Animals and experimental design

All work performed on animals was performed according to the 2010/63/EU of the European Parliament and of the Council of 22 September 2010 and has been approved by local authorities (Landesamt für Natur, Umwelt und Verbraucherschutz Nordrhein-Westfalen; approval IDs: 84-02.05.20.13.097, 84-02.05.40.14.046, 84-02.05.50.17.006, 81-02.05.50.17.019 and 84-02.04.2015.A585). Studies were conducted strictly following the ARRIVE guidelines [61]. C57BL6J and NOX4^−/−^ mice were used for all experiments, were group-caged, and kept in a 12 h light/dark cycle. Food and water were available ad libitum. General experimental toxic demyelination was induced by feeding the mice (8–12 weeks of age at the beginning of the experiment) a diet containing 0.2% cuprizone (bis-cyclohexanone oxaldihydrazone; Sigma-Aldrich) mixed into a ground standard rodent chow [30, 62]. The cuprizone diet was maintained for 5 weeks. Interruption of the cuprizone diet promotes spontaneous remyelination [30, 63] and, therefore, we tested another two groups of animals 7 and 25 days after reintroduction of normal food.

NOX4^−/−^ and WT mice in the cuprizone model were tested in vitro (gene expression), ex vivo (ROS and MCB imaging) and in vivo (single unit analysis). The effect of the cuprizone diet on NOX4^−/−^ mice was analyzed at baseline [normal food (B)], at the end of the cuprizone diet [full demyelination (D)], early remyelination [7 days after remyelination (7dR)] and full remyelination [25 days after remyelination (25dR)].

### Preparation of brain slices for live cell imaging

Brain slices containing the hippocampal circuits were obtained as described previously [64]. Briefly, mice (8–12 weeks of age) were deeply anesthetized with isoflurane (4% in O_2_) and decapitated. Brains were quickly removed and glued on an agar block which was placed on a vibratome (Leica, Germany) and superfused with ice-cold aCSF containing the following in mM: sucrose, 200; glucose, 10; PIPES, 20; KCl, 2.5; MgSO_4_, 10; CaCl_2_, 0.5; pH 7.35 with NaOH. Coronal slices (100 µm) were then cut and transferred to a holding chamber (oxygenated aCSF, 31 °C) and allowed to rest for 60 min before the experiments started.

### Stereotaxic surgery and electrode implantation

For the recording of spontaneous unit activities in freely behaving mice, animals were anesthetized using isoflurane (3% in O_2_; Abbot). All pressure points were covered with 2% xylocaine gel (Astra Zeneca) and subcutaneous tissues were infused with 2% xylocaine solution to induce local anesthesia. Before performing surgery, an additional dose of carprofen (Rimadyl, 5 mg/kg) was administered to relieve post-operatory pain. Microwire arrays (one array, eight electrodes and one reference/array; EMB, 39116 Magdeburg, Germany) were implanted unilaterally (left hemisphere) under stereotaxic control (David Kopf Instruments, USA) into CA1 using the following stereotaxic coordinates [65]: anteroposterior, − 2.18 mm; lateral, 2.00 mm from bregma, and dorsoventral, 1.25 mm from the brain surface as previously described [66, 67]. An additional ground electrode was placed in proximity of the midline over the cerebellar region in the right hemisphere. At the end of the experiments, animals were killed by an overdose of isoflurane (3% in O_2_; Abbot) and brains were rapidly removed and fixed in 4% phosphate-buffered formaldehyde. Electrode positions were identified in 50 µM cresyl violet-counterstained frontal brain sections.

### In vivo electrophysiological recordings and single unit analysis

Single unit activities were recorded using a Multichannel Amplifier System (Alpha Omega, Israel). A surgical recovery period of 7–10 days was given to all animals before the experiment. Signals were band-pass filtered at 100 Hz to 20 kHz and processed at sampling rate of 40 kHz. Spike of individual neurons was sorted by time–amplitude window discrimination and principal component analysis (Offline Sorter, Plexon Inc., Dallas, TX, USA) and validated through quantification of cluster separation, as previously described [67]. Baseline firing rates of sorted neurons were analyzed in 1 s segments (1 bin), with a custom-built interface in NeuroExplorer (Plexon).

### Slice preparations for intracellular recordings

NOX4^−/−^ and WT mice (8–12 weeks) were depicted as described before and transferred to ice-cold aCSF. Combined amygdala-hippocampus-neocortex slices with a thickness of 500 µm were obtained using a vibratome (Integraslice 7550 PSDS, Campden Instruments, Loughborough) and transferred directly to a preincubation bath for 60 min. Slices were kept in aCSF (124 mM NaCl, 4 mM KCl, 1.24 mM NaH_2_PO_4_, 26 mM NaHCO_3_, 10 mM glucose, 1 mM CaCl_2_, 1.3 mM MgSO_4_, pH 7.35) that was gassed with Carbogen (95% O_2_, 5% CO_2_) and preheated to 28 °C. After 30 min CaCl_2_ level was increased to 2 mM (aCSF II) for Neuron stabilization and to prevent cell injury during the electrode adjustment. Slices were then transferred on a transparent membrane and placed in a recording chamber that was continuously perfused with carbonated aCSF (2 ml/min, 32 °C) and additionally Carbogen was evaporated directly over the surface of the slices to guarantee an optimal oxygen supply. This kept the chamber humid and provided the neurons with an adequate supply of nutrients. A light microscope (Zeiss), which was placed above the interface chamber, was used for positioning the electrodes. To reduce disruptive factors the chamber was placed on a metallic anti-vibration table and a Faraday cage shielded it from electrical interference.

### Intracellular signal

To measure the membrane potential of individual cells, electrodes were put into the stratum pyramidale in CA1 to achieve the highest possible probability of encountering large cell somata (especially pyramidal neurons) in this layer. Intracellular electrodes were filled with a 2 M potassium methyl sulfate solution (120 mM CS-gluconate, 5 mM CsCl, 10 mM TEA-Cl, 8 mM NaCl, 10 mM Hepes, 5 mM EDTA, 4 mM MgATP, 0.3 mM Na_3_GTP and 5 mM QX-3149) and the resistance was between 75 and 150 mΩ. A chlorinated silver wire half inserted into the electrode was used to connect the active bridge mode amplifier. Current injections (Ci) triggered action potentials (AP), and an analog-to-digital converter (Digidata 1322 A, Axon Instruments) visualized the initially analog information transmissions of the registered output signals and analyzed it via AxoScope software (V10.1, Axon Instruments). The recorded parameters were, the depolarization (dep) and the amplitude and duration after hyperpolarization (AHP.a, AHP.d). APs that were recorded after triggered by Ci were analyzed as the amplitude of the first AP (Ci.amp) and the interval between the first and second AP (Ci.isi).

### Statistics

Prior to statistical analysis all data were tested for normal distribution and detection of outliers. Data were statistically processed using two-tailed Student’s t-test with two group comparisons. Comparisons between multiple groups was tested by one-way analysis of variance (ANOVA) followed by Tukey’s post hoc. Statistical significances were reached when *p < 0.05, **p < 0.01, ***p < 0.001 or ****p < 0.0001. All data are given as mean ± SEM. Graphpad prism (V8.0.2, GraphPad Software Inc., CA, USA) was used for statistical analysis. Analysis for live cell Imaging raw data was done by Metafluor Fluorescence Ratio Imaging Software (Molecular Devices, LLC, Canada/US) followed by plotting the data with Origin (V2019, OriginLab Corporation, Northampton, MA, USA) and ImageJ (V1.46r, Wayne Rasband, National Institute of Health, USA). Clustering of significantly regulated proteins was performed using Progenesis (Nonlinear Diagnostics). GO analysis was done by using Panther classification system version 16. Data were illustrated by using Graphpad prism and Adobe illustrator (v2020,Adobe Systems Software Ireland Limited, Dublin, Republic of Ireland).

## Supplementary Information

Below is the link to the electronic supplementary material.

Supplementary file1 (PDF 608 KB)

Supplementary file2 (PDF 368 KB)

## Data Availability

The datasets used and analyzed during the current study are available from the corresponding author on reasonable request.
