# Supplementary material for: NOX4-derived ROS are neuroprotective by balancing intracellular calcium stores
Source: Cell Mol Life Sci. 2023 Apr 21;80(5):127. doi: 10.1007/s00018-023-04758-z (PMC10119225; doi:10.1007/s00018-023-04758-z)
Supplement: Supplementary file 1 — Supplementary file1 (PDF 608 KB) [file 18_2023_4758_MOESM1_ESM.pdf]

Highest mean condition	Lowest mean condition	Mass	Description	WT_1a	WT_1b	WT_1c	WT_2a	WT_2b	WT_2c	WT_3a	WT_3b	WT_3c	WT_4a	WT_4b	WT_4c	NOX4_1a	NOX4_1b	NOX4_1c	NOX4_2a	NOX4_2b	NOX4_2c	NOX4_3a	NOX4_3b	NOX4_3c	NOX4_4a	NOX4_4b	NOX4_4c
NOX4	WT	98077	Uncharacterized protein (Fragment) OS=Mus musculus OX=10090 GN=Sptan1 PE=2 SV=1	832	773	795	821	780	824	777	921	802	619	834	389	783	1092	874	880	928	1251	904	905	980	716	872	936
NOX4	WT	67968	Syntaxin-binding protein 1 OS=Mus musculus OX=10090 GN=Stxbp1 PE=1 SV=2	19226	28747	33454	23814	25634	26322	19510	21543	22352	16632	23784	25448	23949	27513	28416	36674	39240	40798	32633	34111	33082	24351	24628	25697
NOX4	WT	223772	Myosin-4 OS=Mus musculus OX=10090 GN=Myh4 PE=2 SV=1	10	0	9	30	125	140	38	44	38	15	13	4	44	25	15	112	89	79	9220	118	80	1591	336	62
NOX4	WT	245943	Ninein OS=Mus musculus OX=10090 GN=Nin PE=1 SV=4	497	252	336	271	298	287	397	449	393	525	428	169	587	630	483	515	530	638	630	634	615	371	527	568
NOX4	WT	214293	Plexin-A1 OS=Mus musculus OX=10090 GN=Plxna1 PE=1 SV=1	4297	3004	3010	2837	3276	3286	3100	3842	4005	4034	3100	4367	3448	4187	4551	3046	4057	4562	3838	5631	5971	3490	3770	4945
NOX4	WT	54685	Calcium/calmodulin-dependent protein kinase type II subunit alpha OS=Mus musculus OX=10090 GN=Camk2a PE=1 SV=2	16188	14801	14470	11455	10715	10867	11365	11727	10865	11436	14846	16149	16612	17366	16919	18297	17901	18010	17985	17230	17625	11069	11691	11318
NOX4	WT	106085	Catenin alpha-2 OS=Mus musculus OX=10090 GN=Ctnna2 PE=1 SV=3	809	730	729	855	796	709	629	551	576	533	652	416	1859	894	628	633	606	610	6868	5487	4441	804	719	804
NOX4	WT	159140	Rho-associated protein kinase 1 OS=Mus musculus OX=10090 GN=Rock1 PE=1 SV=1	5326	5452	5512	4350	8821	8613	4912	5049	5199	4083	4159	3958	9424	10560	10746	9761	9419	9394	16054	14190	14934	8104	7309	7601
NOX4	WT	137612	Golgi apparatus protein 1 OS=Mus musculus OX=10090 GN=Glg1 PE=1 SV=1	2842	2391	2248	2997	3057	3029	3981	3822	3942	2897	1897	2112	4232	4206	4272	4386	4182	4341	2866	3191	3132	3432	3254	3316
NOX4	WT	214785	Plexin-A2 OS=Mus musculus OX=10090 GN=Plxna2 PE=1 SV=2	214	76	141	728	4904	5930	561	2681	2329	1018	1449	1143	812	6874	6810	478	2557	2705	4246	4196	3569	2661	2702	1679
NOX4	WT	98942	Band 4.1-like protein 1 OS=Mus musculus OX=10090 GN=Epb41l1 PE=1 SV=2	167	195	185	267	302	314	163	331	325	174	221	33	304	356	359	258	306	495	328	499	583	279	424	499
NOX4	WT	205777	Centriolin OS=Mus musculus OX=10090 GN=Cntrl PE=1 SV=2	510	482	385	219	512	499	247	268	300	351	523	512	254	372	354	339	928	790	414	785	704	1252	912	534
NOX4	WT	176933	Eukaryotic translation initiation factor 4 gamma 1 OS=Mus musculus OX=10090 GN=Eif4g1 PE=1 SV=1	405	508	582	83	150	162	156	157	172	213	160	228	279	548	551	214	365	404	750	574	315	248	154	180
NOX4	WT	141955	Structural maintenance of chromosomes protein 3 OS=Mus musculus OX=10090 GN=Smc3 PE=1 SV=2	49	27	55	116	884	462	74	250	112	54	82	10	290	3041	335	237	582	120	149	139	79	199	208	215
NOX4	WT	104790	AP-1 complex subunit beta-1 OS=Mus musculus OX=10090 GN=Ap1b1 PE=1 SV=2	226	339	135	202	350	350	294	340	375	283	175	117	300	315	373	453	585	528	194	295	199	364	396	387
NOX4	WT	182822	DNA topoisomerase 2-beta OS=Mus musculus OX=10090 GN=Top2b PE=1 SV=2	0	6	0	49	95	109	214	178	187	218	43	26	157	195	180	82	86	60	267	255	390	126	107	82
NOX4	WT	106413	Lon protease homolog_ mitochondrial OS=Mus musculus OX=10090 GN=Lonp1 PE=1 SV=2	2491	2546	2061	2351	900	837	1595	924	850	1912	1907	1907	1995	1996	2132	2144	2100	1948	2811	2675	2726	1785	1579	1777
NOX4	WT	211494	Plexin A3 OS=Mus musculus OX=10090 GN=Plxna3 PE=4 SV=1	71	130	129	174	204	238	147	168	191	132	110	57	153	181	285	145	155	219	185	240	230	244	282	312
NOX4	WT	95238	Heat shock 70 kDa protein 4L OS=Mus musculus OX=10090 GN=Hspa4l PE=1 SV=2	1470	1743	1646	2914	2469	2493	1390	1266	1427	1329	1609	2073	1663	1835	1968	2203	2185	2340	2138	1858	3234	2667	2765	2919
NOX4	WT	136083	Catenin delta-2 OS=Mus musculus OX=10090 GN=Ctnnd2 PE=1 SV=1	100	160	175	617	433	483	517	316	470	205	288	116	444	439	424	455	350	263	790	756	659	348	309	261
NOX4	WT	212224	Unconventional myosin-Vb OS=Mus musculus OX=10090 GN=Myo5b PE=1 SV=2	269	84	168	148	157	201	151	253	295	226	277	53	204	367	273	186	258	424	330	457	505	302	419	554
NOX4	WT	103794	Band 4.1-like protein 3 OS=Mus musculus OX=10090 GN=Epb41l3 PE=1 SV=1	29	0	22	687	1259	1065	742	685	798	328	401	276	1099	1302	1432	1506	1452	1248	568	375	349	1074	994	793
NOX4	WT	181602	Mitogen-activated protein kinase kinase kinase 4 OS=Mus musculus OX=10090 GN=Map3k4 PE=1 SV=2	0	24	0	912	176	182	517	30	37	336	33	4	306	75	766	575	580	565	582	591	695	987	978	999
NOX4	WT	181976	GATOR complex protein DEPDC5 OS=Mus musculus OX=10090 GN=Depdc5 PE=1 SV=2	0	0	10	445	397	325	315	234	224	73	100	44	282	368	390	773	712	650	132	94	60	303	321	233
NOX4	WT	237765	Dedicator of cytokinesis protein 9 OS=Mus musculus OX=10090 GN=Dock9 PE=1 SV=2	129	435	842	108	148	136	77	93	121	110	114	63	153	249	197	205	253	269	238	502	363	238	206	129
NOX4	WT	92137	Clathrin coat assembly protein AP180 OS=Mus musculus OX=10090 GN=Snap91 PE=1 SV=1	6900	6479	5941	15421	14530	14590	10766	7751	10735	5168	7004	5994	11313	14026	14074	17154	18268	17663	7921	8159	7998	14922	14652	13886
NOX4	WT	248210	Dedicator of cytokinesis protein 10 OS=Mus musculus OX=10090 GN=Dock10 PE=1 SV=3	74	141	107	32	44	79	53	26	33	61	187	211	136	112	104	146	194	130	183	343	344	72	104	76
NOX4	WT	176281	Tetratricopeptide repeat domain 37 OS=Mus musculus OX=10090 GN=Ttc37 PE=1 SV=1	210	103	210	1486	603	461	659	208	448	254	329	309	674	545	374	955	617	502	869	815	700	619	483	441
NOX4	WT	234566	Ankyrin repeat domain 12 OS=Mus musculus OX=10090 GN=Ankrd12 PE=1 SV=1	101	38	45	82	103	105	116	137	124	93	15	23	145	143	127	157	149	139	56	59	49	153	149	138
NOX4	WT	228318	Dedicator of cytokinesis protein 4 OS=Mus musculus OX=10090 GN=Dock4 PE=1 SV=1	138	0	117	610	506	420	251	245	171	0	0	0	304	298	289	652	668	667	193	89	18	355	404	324
NOX4	WT	63513	5'-nucleotidase domain-containing protein 3 OS=Mus musculus OX=10090 GN=Nt5dc3 PE=1 SV=1	767	726	1703	607	771	733	725	902	784	946	1255	1304	905	1096	1096	1376	1536	1719	1369	1473	1472	811	881	858

[illegible]

WT	NOX4	36841	L-lactate dehydrogenase A chain OS=Mus musculus OX=10090 GN=Ldha PE=1 SV=3	25065	18299	24031	15309	16736	15472	11698	12682	11990	14148	11559	16608	11806	12462	12381	13221	13831	13533	12419	14238	15288	11440	11613	11677
WT	NOX4	79995	Peroxisomal multifunctional enzyme type 2 OS=Mus musculus OX=10090 GN=Hsd17b4 PE=1 SV=3	4520	3873	2993	2766	2499	2641	3117	2607	2663	3950	4387	4722	2768	2171	2219	2125	1935	1710	3623	3557	2892	2735	2539	2402
WT	NOX4	54786	Dihydrolipoyl dehydrogenase_ mitochondrial OS=Mus musculus OX=10090 GN=Dld PE=1 SV=2	17748	22800	23496	14611	16458	16398	12806	13867	14136	11627	15443	17046	12003	12813	12802	15709	15790	17455	10494	9327	9745	13365	14033	14999
WT	NOX4	51672	Trifunctional enzyme subunit beta_ mitochondrial OS=Mus musculus OX=10090 GN=Hadhb PE=1 SV=1	6665	7172	5720	6394	6580	6832	5141	5910	6265	6653	7027	6461	5066	5168	5399	3774	3449	3654	5460	5794	5477	5194	5054	5364
WT	NOX4	48307	Long-chain specific acyl-CoA dehydrogenase_ mitochondrial OS=Mus musculus OX=10090 GN=Acadl PE=1 SV=2	3152	3228	2887	7027	6564	6846	6385	6512	7151	6449	6831	8301	4844	4683	4611	1969	1876	1914	4581	4384	4390	4737	4724	4870
WT	NOX4	87794	Golgin subfamily A member 1 OS=Mus musculus OX=10090 GN=Golga1 PE=1 SV=2	2489	2685	2267	1544	1106	1142	1299	883	1063	1512	1944	2349	980	803	862	1392	1192	1254	1048	910	1061	942	807	771
WT	NOX4	129986	VPS10 domain-containing receptor SorCS2 OS=Mus musculus OX=10090 GN=Sorcs2 PE=1 SV=2	15108	15918	17384	10591	11276	11268	10503	11557	11463	6746	7433	8985	8922	8892	9826	8320	9268	9993	7312	8550	8397	7906	8615	9472
WT	NOX4	193595	Ankyrin repeat domain-containing protein 26 OS=Mus musculus OX=10090 GN=Ankrd26 PE=1 SV=1	2052	571	1850	1003	1014	960	1479	1246	1270	1862	1381	1342	1176	1117	1117	805	823	794	1016	871	913	1246	970	1097
WT	NOX4	97919	Glycogen phosphorylase_ liver form OS=Mus musculus OX=10090 GN=Pygl PE=1 SV=4	1088	633	967	1033	842	1314	797	624	833	865	855	1080	768	491	510	535	398	276	747	1012	852	507	743	590
WT	NOX4	99565	Cytosolic 10-formyltetrahydrofolate dehydrogenase OS=Mus musculus OX=10090 GN=Aldh1l1 PE=1 SV=1	2510	2520	1579	2419	2310	2452	1978	2154	1999	1737	1644	2820	1788	1578	1458	999	1019	748	1963	1512	1346	1627	1249	1495
WT	NOX4	57051	Aldehyde dehydrogenase_ mitochondrial OS=Mus musculus OX=10090 GN=Aldh2 PE=1 SV=1	6634	6857	6840	12177	12470	12778	11758	12762	12300	13412	16704	18312	7490	7941	7903	3050	3196	3208	7277	7758	7783	10088	10130	10902
WT	NOX4	59328	Serine/threonine-protein phosphatase 2B catalytic subunit alpha isoform OS=Mus musculus OX=10090 GN=Ppp3ca PE=1 SV=1	10724	10843	9834	6345	6109	6162	5481	5775	5463	7406	8204	10702	6476	6186	6169	6305	6234	6521	6451	6321	6263	5640	5314	5944
WT	NOX4	74523	Prelamin-A/C OS=Mus musculus OX=10090 GN=Lmna PE=1 SV=2	631	422	479	674	566	520	666	538	533	883	734	881	547	452	396	309	275	243	721	714	740	408	405	423
WT	NOX4	47074	Isocitrate dehydrogenase [NADP] cytoplasmic OS=Mus musculus OX=10090 GN=Idh1 PE=1 SV=2	3985	4056	4206	2438	2479	2321	1975	1954	1856	1802	2385	5029	1813	1802	1820	2625	2537	2611	1935	2265	1739	2080	2051	2085
WT	NOX4	176202	Eukaryotic translation initiation factor 4 gamma 3 OS=Mus musculus OX=10090 GN=Eif4g3 PE=1 SV=2	6376	6378	5649	3311	2648	2512	3725	3104	3789	3283	4176	4394	2837	2552	2696	2689	2687	3335	3842	2802	2739	3120	2254	2994
WT	NOX4	143767	Rho GTPase-activating protein 29 OS=Mus musculus OX=10090 GN=Arhgap29 PE=1 SV=1	1395	1140	1201	772	658	605	1161	1249	1236	1355	1585	1485	1129	1089	1293	768	686	747	708	751	718	635	593	544
WT	NOX4	211773	Chromodomain-helicase-DNA-binding protein 2 OS=Mus musculus OX=10090 GN=Chd2 PE=1 SV=1	1602	1527	1723	1287	1411	1362	1300	1499	1511	1594	1487	1707	1295	1493	1488	744	869	957	1211	1433	1294	1171	1288	1347
WT	NOX4	192111	MKIAA0051 protein (Fragment) OS=Mus musculus OX=10090 GN=Iqgap1 PE=2 SV=1	4064	4739	3742	1986	4324	5013	2871	5044	4946	3125	5144	4565	2803	2801	2729	2974	2525	2328	3458	2997	3122	4662	4040	3396
WT	NOX4	58580	T-complex protein 1 subunit delta OS=Mus musculus OX=10090 GN=Cct4 PE=1 SV=3	2425	2135	2127	1235	1195	1084	1021	896	1019	1474	1383	2066	986	976	1144	1201	1212	1251	1028	1009	1083	1001	1151	1137
WT	NOX4	226238	E3 ubiquitin-protein ligase TRIP12 OS=Mus musculus OX=10090 GN=Trip12 PE=1 SV=1	797	546	674	829	419	363	781	526	576	873	436	1357	1004	404	473	521	156	143	214	412	303	364	305	523
WT	NOX4	45577	Fructose-bisphosphate aldolase OS=Mus musculus OX=10090 GN=Aldoa PE=1 SV=1	292	64	102	225	170	166	92	144	134	170	49	702	95	101	142	69	76	76	52	48	44	107	117	148
WT	NOX4	41059	Guanine nucleotide-binding protein G(i) subunit alpha-2 OS=Mus musculus OX=10090 GN=Gnai2 PE=1 SV=5	7824	8778	8943	5517	5991	5941	6068	6282	6485	9037	11177	12958	5226	5594	5746	3137	3173	3461	7968	8268	8585	6007	5986	6040
WT	NOX4	55374	Keratin_ type II cytoskeletal 74 OS=Mus musculus OX=10090 GN=Krt74 PE=3 SV=1	2080	1709	1449	945	741	712	797	683	789	965	1170	1451	712	660	749	581	885	937	938	895	1030	765	720	873
WT	NOX4	57136	4-aminobutyrate aminotransferase_ mitochondrial OS=Mus musculus OX=10090 GN=Abat PE=1 SV=1	5895	5577	6215	7220	6787	6751	6229	6087	6177	5398	6330	7519	4684	4405	4773	2859	2814	2615	4433	4758	5084	4843	4594	4751
WT	NOX4	189821	Intersectin-2 OS=Mus musculus OX=10090 GN=Itsn2 PE=1 SV=2	267	348	283	111	97	122	160	16	42	267	128	193	194	108	41	206	183	41	47	112	17	28	23	19
WT	NOX4	205529	Proteasome adapter and scaffold protein ECM29 OS=Mus musculus OX=10090 GN=Ecpas PE=1 SV=3	202	155	243	133	128	103	127	156	132	327	334	979	214	132	129	57	94	55	212	203	168	79	63	63
WT	NOX4	47799	Flotillin-1 OS=Mus musculus OX=10090 GN=Flot1 PE=1 SV=1	1319	1348	1422	2568	2184	1999	1835	1514	1243	1074	1837	1810	1432	1377	1381	1404	1426	1301	1191	875	1471	1548	1492	1487
WT	NOX4	76780	Nucleolin OS=Mus musculus OX=10090 GN=Ncl PE=1 SV=2	1069	1121	1081	1658	1590	1599	2429	2444	2404	1382	1713	1799	1718	1559	1493	973	855	531	1133	1230	1141	1526	1598	1525
WT	NOX4	35924	Apolipoprotein E OS=Mus musculus OX=10090 GN=Apoe PE=1 SV=2	1660	1712	1670	3256	2908	2761	1904	1687	1553	2475	2177	3181	1502	1436	1450	816	733	682	1759	1326	1327	2107	2162	2208
WT	NOX4	72585	Polyadenylate-binding protein OS=Mus musculus OX=10090 GN=Pabpc4 PE=1 SV=1	92	171	285	129	163	211	44	55	55	24	16	16	27	25	30	15	16	8	38	84	72	54	69	60
WT	NOX4	73027	Probable ATP-dependent RNA helicase DDX17 OS=Mus musculus OX=10090 GN=Ddx17 PE=1 SV=1	1025	734	787	521	553	530	562	599	539	631	586	616	569	588	547	390	424	416	622	551	480	458	427	473
WT	NOX4	36858	L-lactate dehydrogenase B chain OS=Mus musculus OX=10090 GN=Ldhb PE=1 SV=2	7936	9082	7619	7792	7921	7272	6137	6756	6361	5781	6776	7816	5970	5837	5861	3790	3647	3788	5199	5432	5442	5197	4832	5204
WT	NOX4	228516	Otoferlin OS=Mus musculus OX=10090 GN=Otof PE=1 SV=1	664	437	381	403	392	350	379	437	349	514	628	610	348	324	222	322	301	255	309	207	153	418	446	367
WT	NOX4	39794	Fructose-bisphosphate aldolase C OS=Mus musculus OX=10090 GN=Aldoc PE=1 SV=4	626	594	430	1267	1239	1214	795	841	863	1131	1151	4477	574	590	680	316	311	334	600	687	660	766	799	789
WT	NOX4	60166	T-complex protein 1 subunit eta OS=Mus musculus OX=10090 GN=Cct7 PE=1 SV=1	1804	2390	2441	1100	5417	5258	964	4454	1152	1044	749	952	734	880	924	1283	1260	1467	934	938	807	949	1005	910
WT	NOX4	71037	Heat shock 70 kDa protein 1-like OS=Mus musculus OX=10090 GN=Hspa1l PE=1 SV=4	375	333	250	254	220	274	367	280	330	306	301	275	270	254	287	243	270	180	161	259	197	264	282	261
WT	NOX4	69861	Heterogeneous nuclear ribonucleoprotein Q OS=Mus musculus OX=10090 GN=Syncrip PE=1 SV=2	4276	3784	3656	4091	4141	3993	4051	4383	4324	3738	4167	4424	3618	3668	3838	2014	1926	1879	3174	3336	3036	3244	3130	3074
WT	NOX4	32705	Triosephosphate isomerase OS=Mus musculus OX=10090 GN=Tpi1 PE=1 SV=4	24798	23499	23334	16350	14948	15218	13596	13737	13184	12469	18499	16870	11650	11239	11686	12549	12202	14043	13627	11763	14585	10742	10740	10755
WT	NOX4	32360	Voltage-dependent anion-selective channel protein 2 OS=Mus musculus OX=10090 GN=Vdac2 PE=1 SV=2	40807	43045	41473	21413	19734	19627	21949	21374	21702	26581	34711	40855	19866	18992	19376	20386	20924	20676	21383	23244	24357	20416	18327	18708
WT	NOX4	54585	Fumarate hydratase_ mitochondrial OS=Mus musculus OX=10090 GN=Fh PE=1 SV=3	5821	6991	6410	4983	5375	5279	4344	4340	4737	3601	5007	6325	3435	3443	3539	2695	2749	2830	3151	4042	3954	4577	4319	4557
WT	NOX4	162102	Roundabout homolog 2 OS=Mus musculus OX=10090 GN=Robo2 PE=1 SV=2	3531	3506	3439	1233	1272	1375	1288	1439	1508	2706	3684	3102	1838	1626	1827	1436	1378	1492	2953	425	623	828	744	596
WT	NOX4	42861	Glutamine synthetase OS=Mus musculus OX=10090 GN=Glul PE=1 SV=6	7849	9024	9519	3205	3053	3138	2276	2293	2131	682	885	784	2228	2446	1983	2221	2173	1926	670	450	320	1255	1131	1170
WT	NOX4	40889	NADH dehydrogenase [ubiquinone] 1 alpha subcomplex subunit 10_ mitochondrial OS=Mus musculus OX=10090 GN=Ndufa10 PE=1 SV=1	5487	10853	8673	5982	6817	6218	4426	5056	4488	4618	4823	5804	4510	4903	4609	4292	4247	4425	4187	3632	4219	5421	5603	5567
WT	NOX4	196164	DNA-directed RNA polymerase I subunit RPA1 OS=Mus musculus OX=10090 GN=Polr1a PE=1 SV=2	908	952	502	1602	1505	1973	897	1067	1149	792	1307	472	691	641	880	495	398	511	696	627	358	1037	1060	1077
WT	NOX4	14045	Histone H2A.J OS=Mus musculus OX=10090 GN=H2afj PE=1 SV=1	58657	65771	59278	185921	197278	192506	162476	189245	187234	95614	110847	121014	121671	123995	123612	62501	69009	73049	6184	6078	4406	100774	100082	110141
WT	NOX4	47437	Flotillin-2 OS=Mus musculus OX=10090 GN=Flot2 PE=1 SV=2	2374	2168	1428	1901	1869	1956	1575	1648	1643	1765	2024	2392	1606	1666	1644	1515	1514	1581	1356	1587	1425	1630	1655	1643
WT	NOX4	46395	60S ribosomal protein L3 OS=Mus musculus OX=10090 GN=Rpl3 PE=1 SV=3	6779	3755	7722	5502	4493	4327	5617	5643	4941	7904	6742	10735	4879	4643	4568	3208	3089	3086	4712	5121	6213	5000	4527	4894
WT	NOX4	69804	Probable ATP-dependent RNA helicase DDX5 OS=Mus musculus OX=10090 GN=Ddx5 PE=1 SV=2	1367	1329	1201	806	845	908	951	1012	988	1193	1314	1426	985	952	806	496	483	392	904	1114	1136	854	761	762
WT	NOX4	87837	Delta-1-pyrroline-5-carboxylate synthase OS=Mus musculus OX=10090 GN=Aldh18a1 PE=1 SV=2	1241	1898	1949	2154	1846	2139	1447	1772	1643	1390	1639	1460	1118	1167	885	663	548	612	1045	921	1133	1310	1760	1807
WT	NOX4	71662	Uncharacterized protein (Fragment) OS=Mus musculus OX=10090 GN=Slc27a1 PE=2 SV=1	277	198	92	1041	1009	904	575	993	505	572	381	963	456	264	306	64	53	90	208	202	105	535	429	423
WT	NOX4	32612	Succinate dehydrogenase [ubiquinone] iron-sulfur subunit_ mitochondrial OS=Mus musculus OX=10090 GN=Sdhb PE=1 SV=1	10880	10505	10427	8328	8666	8518	7021	7154	7405	7871	8568	8989	6740	6702	6945	6669	6745	6773	7842	8174	8470	7294	7303	7168
WT	NOX4	28946	Phosphoglycerate mutase 1 OS=Mus musculus OX=10090 GN=Pgam1 PE=1 SV=3	19338	18188	15922	8340	8405	8040	8616	8687	8547	9968	9375	11048	8132	8379	8676	7944	7853	7967	9164	9979	10826	6263	6858	7361
WT	NOX4	31874	Enoyl-CoA hydratase_ mitochondrial OS=Mus musculus OX=10090 GN=Echs1 PE=1 SV=1	5105	5167	5216	4338	4618	4616	4366	4702	4620	4767	5337	5622	3782	3958	3820	2996	3000	3542	3515	3573	3246	3449	3526	3929
WT	NOX4	35352	Electron transfer flavoprotein subunit alpha_ mitochondrial OS=Mus musculus OX=10090 GN=Etfa PE=1 SV=2	6925	6568	7076	6452	6241	6348	6209	6098	6334	6667	7149	8562	4734	4307	4949	3370	3346	3370	5755	5585	5511	4784	4977	4862
WT	NOX4	30148	60S ribosomal protein L7a OS=Mus musculus OX=10090 GN=Rpl7a PE=1 SV=2	18858	17147	16290	12681	12133	12292	13043	13720	14011	13627	18459	22883	10686	11147	11407	8217	8270	9276	12907	12723	12200	11177	11493	12106
WT	NOX4	151430	Nephrocystin-3 OS=Mus musculus OX=10090 GN=Nphp3 PE=1 SV=2	982	523	518	852	818	723	606	794	751	805	922	999	557	515	622	220	159	141	593	551	653	651	717	738
WT	NOX4	31516	40S ribosomal protein S2 OS=Mus musculus OX=10090 GN=Rps2 PE=1 SV=3	3770	4725	4133	4471	4520	4730	4967	5243	5187	4774	5285	4992	4418	4545	4359	2548	2580	2363	4297	3917	4184	4501	4514	4759
WT	NOX4	51261	Heterogeneous nuclear ribonucleoprotein K OS=Mus musculus OX=10090 GN=Hnrnpk PE=1 SV=1	5255	2908	3474	2118	1896	1920	1832	2073	1984	2739	2993	3219	1993	1963	1953	1422	1323	1378	2897	2634	2824	1611	1558	1675
WT	NOX4	48754	Ornithine aminotransferase_ mitochondrial OS=Mus musculus OX=10090 GN=Oat PE=1 SV=1	6144	6387	5969	5524	5287	4773	7885	7776	7594	11649	13947	14727	5647	5738	5581	3193	2704	2936	7215	8115	7603	5547	5322	5437
WT	NOX4	162055	Early endosome antigen 1 OS=Mus musculus OX=10090 GN=Eea1 PE=1 SV=2	271	232	386	381	229	210	303	235	205	320	294	634	204	191	157	222	80	84	305	267	322	126	105	101
WT	NOX4	29718	Myristoylated alanine-rich C-kinase substrate OS=Mus musculus OX=10090 GN=Marcks PE=1 SV=2	6220	6022	6600	4185	3687	3576	4554	4722	4917	6076	6437	6233	4801	4719	4766	2502	2670	2742	5748	5999	6133	3438	3358	3791
WT	NOX4	110692	Poly(ADP-ribose) glycohydrolase OS=Mus musculus OX=10090 GN=Parg PE=1 SV=2	248	183	193	372	230	145	283	242	200	313	296	263	254	197	186	192	189	213	253	199	194	167	149	124
WT	NOX4	47700	Saccharopine dehydrogenase-like oxidoreductase OS=Mus musculus OX=10090 GN=Sccpdh PE=1 SV=1	2341	2491	2338	1189	1086	1061	1343	2083	908	1405	1331	1603	1254	1111	934	1269	1173	1032	1281	1077	1102	970	919	883
WT	NOX4	71715	Phosphatidylinositol-binding clathrin assembly protein OS=Mus musculus OX=10090 GN=Picalm PE=1 SV=1	12702	17434	18567	23842	23791	24441	18375	18762	18758	14915	20898	24499	15833	17062	17737	17271	16970	19352	10533	11008	10578	15117	16060	16290
WT	NOX4	47439	60S ribosomal protein L4 OS=Mus musculus OX=10090 GN=Rpl4 PE=1 SV=3	4090	3831	3706	3601	4354	4448	4846	5364	5481	5528	6706	7746	4411	4293	4290	2285	2309	2218	3661	3571	3347	5014	4798	5308
WT	NOX4	38101	Erlin-2 OS=Mus musculus OX=10090 GN=Erlin2 PE=1 SV=1	2598	2725	2130	1720	1554	1498	1444	1523	1387	2388	2839	3497	1136	1248	1152	1084	1124	1191	2421	2356	2166	1876	1747	1777
WT	NOX4	66994	Apoptosis-inducing factor 1_ mitochondrial OS=Mus musculus OX=10090 GN=Aifm1 PE=1 SV=1	1463	1400	1468	1332	1200	1213	1479	1310	1386	1784	2232	2521	1298	1230	1268	585	531	558	1563	1708	1704	1210	1197	1152
WT	NOX4	55413	Mitochondrial-processing peptidase subunit beta OS=Mus musculus OX=10090 GN=Pmpcb PE=1 SV=1	6699	6701	7137	9608	9997	9216	6915	8208	7744	6155	8537	9318	6082	6579	6397	6008	5874	6125	4964	5898	5779	6355	6002	4677
WT	NOX4	68946	Electron transfer flavoprotein-ubiquinone oxidoreductase_ mitochondrial OS=Mus musculus OX=10090 GN=Etfdh PE=1 SV=1	518	490	880	1189	1164	1098	822	733	638	881	371	657	614	557	599	461	474	518	216	230	538	805	762	730
WT	NOX4	22648	40S ribosomal protein S9 OS=Mus musculus OX=10090 GN=Rps9 PE=1 SV=3	7233	6447	5446	6513	6015	5310	6845	6859	7147	8000	9348	11521	5726	5557	5458	3528	3526	3148	6450	6192	7055	6831	6475	6815
WT	NOX4	93619	Protein bicaudal D homolog 2 OS=Mus musculus OX=10090 GN=Bicd2 PE=1 SV=1	261	387	223	251	253	261	283	262	144	247	235	198	257	215	251	239	202	174	180	202	196	179	124	84
WT	NOX4	34471	Actin-related protein 2/3 complex subunit 2 OS=Mus musculus OX=10090 GN=Arpc2 PE=1 SV=3	1655	2256	2985	1959	1874	1642	1387	937	1324	1302	1363	1669	1323	1349	1498	1231	1333	1401	1166	938	1407	1392	1245	1097
WT	NOX4	46725	Isovaleryl-CoA dehydrogenase_ mitochondrial OS=Mus musculus OX=10090 GN=Ivd PE=1 SV=1	2833	2933	2775	3021	2869	2983	2813	2563	2691	2197	1864	3428	2136	1777	1885	1555	1322	1328	2369	3095	2312	2157	2267	2289
WT	NOX4	71163	Uncharacterized protein OS=Mus musculus OX=10090 GN=Hnrnpr PE=2 SV=1	1085	1131	1106	992	1018	989	947	965	971	1016	1070	1042	846	877	919	478	458	407	811	937	927	787	791	849
WT	NOX4	27851	Electron transfer flavoprotein subunit beta OS=Mus musculus OX=10090 GN=Etfb PE=1 SV=3	1352	708	1380	1859	1570	1440	1734	1630	1484	1717	1839	2346	1246	1010	1150	654	481	617	1531	1456	1541	1216	1082	1232
WT	NOX4	178988	Dynamin-binding protein OS=Mus musculus OX=10090 GN=Dnmbp PE=1 SV=2	1394	1140	1190	136	165	146	455	309	325	1404	676	216	679	845	825	227	259	245	2	0	5	35	20	30
WT	NOX4	59539	Keratin_ type II cytoskeletal 73 OS=Mus musculus OX=10090 GN=Krt73 PE=1 SV=1	2877	3364	4128	0	7	0	0	0	0	26	0	8	154	157	215	555	774	687	48	118	47	270	302	340
WT	NOX4	108701	Trifunctional purine biosynthetic protein adenosine-3 OS=Mus musculus OX=10090 GN=Gart PE=1 SV=3	7691	6282	8113	3570	4330	4138	4458	4580	4381	5777	5171	4229	4079	4391	4092	3734	3832	4077	4566	5142	5382	3708	3256	4161
WT	NOX4	16086	40S ribosomal protein S19 OS=Mus musculus OX=10090 GN=Rps19 PE=1 SV=3	1490	877	987	800	887	732	1440	1313	1053	1606	1447	2200	1205	1058	1155	617	457	544	1148	1299	1099	848	683	797
WT	NOX4	37500	Sideroflexin-5 OS=Mus musculus OX=10090 GN=Sfxn5 PE=1 SV=2	1218	1081	1076	948	959	1008	1003	965	992	1042	1123	1259	966	821	927	462	450	581	555	520	740	701	865	927
WT	NOX4	40518	Coxsackievirus and adenovirus receptor homolog OS=Mus musculus OX=10090 GN=Cxadr PE=1 SV=1	9423	7700	15963	4551	4303	4308	4656	3343	3799	4993	11965	8835	4770	3837	4870	4651	4469	4370	4481	4194	5071	4258	4024	4227
WT	NOX4	68635	Acyl-CoA synthetase family member 2_ mitochondrial OS=Mus musculus OX=10090 GN=Acsf2 PE=1 SV=1	778	702	729	1117	1065	1098	1641	1633	1626	2081	1942	2321	1086	1047	1027	278	268	272	934	1001	842	913	850	925
WT	NOX4	101371	MCG133388_ isoform CRA_c OS=Mus musculus OX=10090 GN=Pcdhga2 PE=2 SV=1	17951	1488	1425	708	612	614	943	531	506	1533	1161	1468	625	547	495	490	431	447	1216	1196	1023	350	366	407
WT	NOX4	44410	3-ketoacyl-CoA thiolase A_ peroxisomal OS=Mus musculus OX=10090 GN=Acaa1a PE=1 SV=1	1805	1546	1401	2889	2864	3004	2537	2644	2919	2442	2662	2888	1723	1609	1667	1432	1312	1458	1862	1836	1584	2974	2914	3097
WT	NOX4	52331	Abl interactor 1 OS=Mus musculus OX=10090 GN=Abi1 PE=1 SV=1	3901	3880	3816	1065	2551	2513	2591	3295	3203	3007	3537	4087	2828	2967	2657	1572	1814	2085	2106	1459	2754	1961	2279	2835
WT	NOX4	41086	ATPase family AAA domain-containing protein 1 OS=Mus musculus OX=10090 GN=Atad1 PE=1 SV=1	813	853	1057	666	520	484	471	561	520	771	1002	772	520	458	355	407	265	378	528	849	628	537	464	384
WT	NOX4	49885	Acyl-coenzyme A thioesterase 2_ mitochondrial OS=Mus musculus OX=10090 GN=Acot2 PE=1 SV=2	862	547	427	1487	1177	1134	1362	2612	859	1182	1277	1137	937	682	609	814	566	426	634	479	394	645	543	579
WT	NOX4	59036	Propionyl-CoA carboxylase beta chain_ mitochondrial OS=Mus musculus OX=10090 GN=Pccb PE=1 SV=2	632	460	536	585	670	552	502	577	505	659	637	802	450	469	424	257	349	396	530	696	613	545	563	572
WT	NOX4	48334	Acyl-Coenzyme A dehydrogenase_ short/branched chain OS=Mus musculus OX=10090 GN=Acadsb PE=2 SV=1	7909	6116	5859	6588	6873	6558	7118	9491	7959	9720	10913	13653	5572	6108	6835	3690	3708	4178	7488	7339	6739	6362	6582	7000
WT	NOX4	34015	Transcriptional activator protein Pur-beta OS=Mus musculus OX=10090 GN=Purb PE=1 SV=3	1348	1125	1339	1434	1406	1240	1046	1003	863	828	997	1455	943	776	794	1109	837	980	592	846	672	1014	739	821
WT	NOX4	172305	Tyrosine-protein kinase BAZ1B OS=Mus musculus OX=10090 GN=Baz1b PE=1 SV=2	866	776	466	985	1009	870	1077	2499	1100	1220	1107	1193	924	958	891	675	539	602	678	679	631	867	790	836
WT	NOX4	94610	Receptor-type tyrosine-protein phosphatase alpha OS=Mus musculus OX=10090 GN=Ptpra PE=1 SV=3	252	595	496	709	514	544	549	461	530	548	695	681	410	361	382	188	175	141	420	465	282	509	546	455
WT	NOX4	108235	Thyroid hormone receptor-associated protein 3 OS=Mus musculus OX=10090 GN=Thrap3 PE=1 SV=1	298	400	376	160	94	75	166	204	184	257	323	293	147	180	147	140	130	152	81	96	363	79	94	153
WT	NOX4	245956	Voltage-dependent L-type calcium channel subunit alpha OS=Mus musculus OX=10090 GN=Cacna1c PE=1 SV=1	4532	4526	4508	1975	1807	1520	2571	2436	2136	3816	5951	6718	2152	1703	1800	1371	1506	1384	3387	3312	2490	1491	1763	1384
WT	NOX4	41908	Mitochondrial carrier homolog 1 OS=Mus musculus OX=10090 GN=Mtch1 PE=1 SV=1	2907	1982	2284	1299	1927	1837	1753	1785	1688	2174	2041	2869	1722	1559	1759	1418	1582	1625	1793	2012	2041	1812	1624	1592
WT	NOX4	86222	DNA-binding protein SATB1 OS=Mus musculus OX=10090 GN=Satb1 PE=1 SV=2	1133	1307	1510	1501	1527	1589	1498	1381	1425	1926	1945	1975	1238	1129	1158	1341	1162	1037	1057	1281	1311	1457	1534	1313
WT	NOX4	162887	Membrane-associated guanylate kinase_ WW and PDZ domain-containing protein 1 OS=Mus musculus OX=10090 GN=Magi1 PE=1 SV=1	19781	18126	20459	9706	9132	8498	12002	10860	12232	9843	10861	14269	9664	10055	10637	9507	10002	10836	10358	11204	11526	8617	8159	10139
WT	NOX4	56272	Serine hydroxymethyltransferase_ mitochondrial OS=Mus musculus OX=10090 GN=Shmt2 PE=1 SV=1	1412	1651	2057	1115	1017	1074	978	1218	1347	1571	1626	2035	1162	1110	1380	942	965	862	1268	1226	860	976	865	1191
WT	NOX4	30187	Microtubule-associated protein_ RP/EB family_ member 1 OS=Mus musculus OX=10090 GN=Mapre1 PE=1 SV=1	1791	2223	2631	1789	1799	1681	1782	1687	1906	1600	1346	2053	1460	1449	1514	1684	1799	1868	622	593	564	1599	1562	1855
WT	NOX4	21950	Peroxiredoxin-2 OS=Mus musculus OX=10090 GN=Prdx2 PE=1 SV=3	26453	27269	26884	11837	11430	10724	11270	11249	11426	13906	13307	19067	10996	11257	11302	11319	11373	11493	13494	15362	14992	10280	10267	10507
WT	NOX4	38822	Mitochondrial amidoxime reducing component 2 OS=Mus musculus OX=10090 GN=Marc2 PE=1 SV=1	1605	1648	1063	1152	838	1060	1081	1240	1271	1130	1887	2638	933	988	877	333	536	540	1377	1696	1638	1105	903	1011
WT	NOX4	243786	Polycystic kidney disease and receptor for egg jelly-related protein OS=Mus musculus OX=10090 GN=Pkdrej PE=2 SV=1	2573	2350	2163	1135	1569	1215	1616	2111	1769	2456	1919	1929	1365	1524	1126	665	599	861	1927	2049	2075	1521	1594	802
WT	NOX4	119053	Kinesin-like protein KIF11 OS=Mus musculus OX=10090 GN=Kif11 PE=1 SV=1	1315	1638	1420	851	889	1110	876	804	1046	1176	1524	1359	763	642	665	788	844	761	504	527	407	868	1018	996
WT	NOX4	101119	Protocadherin gamma A12 OS=Mus musculus OX=10090 GN=Pcdhga12 PE=1 SV=1	172	70	70	79	134	116	143	122	111	187	190	223	118	132	96	45	62	14	105	54	79	93	65	93
WT	NOX4	25082	Transmembrane emp24 domain-containing protein 10 OS=Mus musculus OX=10090 GN=Tmed10 PE=1 SV=1	5897	6727	6209	4192	4201	4626	4349	4784	4684	6331	7565	8609	4379	4432	4395	3900	4044	4054	5331	5484	5400	4714	4520	5166
WT	NOX4	180893	E3 ubiquitin-protein ligase HECW1 OS=Mus musculus OX=10090 GN=Hecw1 PE=1 SV=3	11264	10989	10415	6062	5548	5852	5323	6422	6392	6940	8727	10272	5886	6666	6906	4838	5491	5797	6916	8463	7443	5079	5415	5391
WT	NOX4	102859	Golgb1 protein OS=Mus musculus OX=10090 GN=Golgb1 PE=2 SV=1	1290	1295	921	676	720	709	677	986	876	1499	1374	1320	797	776	804	741	681	729	1069	999	888	716	522	786
WT	NOX4	98944	Janus kinase and microtubule-interacting protein 3 OS=Mus musculus OX=10090 GN=Jakmip3 PE=1 SV=2	2291	1805	1680	1576	1676	1669	1107	1249	1163	1765	1769	1956	1161	1138	1224	1102	1094	1127	1357	1553	1523	1334	1285	1208
WT	NOX4	178555	MKIAA0559 protein (Fragment) OS=Mus musculus OX=10090 GN=Pclo PE=2 SV=1	4893	4136	3975	3084	2379	2037	3524	3356	3203	4838	5618	6208	2820	2709	2863	1574	1672	1999	4109	3865	3062	2519	2446	2296
WT	NOX4	50656	2-oxoisovalerate dehydrogenase subunit alpha_ mitochondrial OS=Mus musculus OX=10090 GN=Bckdha PE=1 SV=1	788	818	924	261	240	335	410	460	431	828	631	498	390	335	276	308	278	117	563	495	513	410	313	244
WT	NOX4	69126	Kinesin light chain 4 OS=Mus musculus OX=10090 GN=Klc4 PE=1 SV=1	655	509	463	359	249	216	257	214	191	349	342	402	218	208	223	257	271	222	217	162	121	248	223	235
WT	NOX4	57384	D-3-phosphoglycerate dehydrogenase OS=Mus musculus OX=10090 GN=Phgdh PE=1 SV=3	526	684	492	566	597	617	493	453	465	327	630	80	317	322	319	282	240	260	5	54	234	255	291	249
WT	NOX4	35075	Gamma-soluble NSF attachment protein OS=Mus musculus OX=10090 GN=Napg PE=1 SV=1	1140	1128	903	1452	1142	1330	1028	1319	1132	959	1483	1585	974	1068	827	1097	1029	1117	921	878	923	1057	992	1007
WT	NOX4	23935	Ras-related protein Rab-5B OS=Mus musculus OX=10090 GN=Rab5b PE=1 SV=1	2058	2256	2258	1932	2002	1855	1725	1904	1905	2148	2510	2769	1825	1902	2037	1547	1696	1868	1478	1489	1452	1679	1713	1594
WT	NOX4	67800	Sodium- and chloride-dependent GABA transporter 1 OS=Mus musculus OX=10090 GN=Slc6a1 PE=1 SV=2	4320	4403	4593	5010	5339	5092	4177	4566	4796	3527	4048	5298	3887	3936	4292	2480	2497	2425	3686	3587	3700	4579	4448	4585
WT	NOX4	185897	Collagen alpha3(VI) (Fragment) OS=Mus musculus OX=10090 GN=Col6a3 PE=2 SV=1	568	491	424	849	667	604	778	418	344	532	499	504	455	371	368	543	428	451	468	353	335	394	347	295
WT	NOX4	171256	DNA topoisomerase 2-binding protein 1 OS=Mus musculus OX=10090 GN=Topbp1 PE=1 SV=2	359	565	604	209	218	185	166	254	196	263	278	340	236	226	178	158	162	226	117	147	278	185	194	238
WT	NOX4	42901	Basigin OS=Mus musculus OX=10090 GN=Bsg PE=1 SV=2	6567	5074	5529	2270	2180	2153	1384	1368	1237	2498	2833	4022	1737	1557	1593	1575	1452	1489	2492	2892	2877	1495	1467	1564
WT	NOX4	35843	Phospholipid phosphatase 3 OS=Mus musculus OX=10090 GN=Plpp3 PE=1 SV=1	2816	3211	3781	3561	3834	4075	4256	4467	4827	6539	9007	10786	2949	2894	3209	784	981	949	3745	4370	4507	3317	3248	3131
WT	NOX4	116764	Coiled-coil domain-containing protein 57 OS=Mus musculus OX=10090 GN=Ccdc57 PE=1 SV=1	273	290	216	36	105	212	523	709	811	609	596	669	458	531	624	25	55	51	130	96	97	117	149	88
WT	NOX4	47523	2'_3'-cyclic-nucleotide 3'-phosphodiesterase OS=Mus musculus OX=10090 GN=Cnp PE=1 SV=3	206	231	91	2174	2457	1810	598	249	200	221	219	109	218	203	143	129	130	95	133	75	54	336	224	107
WT	NOX4	48447	Keratin_ type I cytoskeletal 17 OS=Mus musculus OX=10090 GN=Krt17 PE=1 SV=3	44	30	39	36	27	20	84	43	24	909	1489	2006	37	26	23	6	6	2	96	87	170	23	9	9
WT	NOX4	136789	SAM and SH3 domain-containing protein 1 OS=Mus musculus OX=10090 GN=Sash1 PE=1 SV=1	4305	4043	4209	45	46	48	18	35	19	323	638	170	161	57	112	99	59	165	28	7	36	16	19	14
WT	NOX4	38525	Heterogeneous nuclear ribonucleoprotein D0 OS=Mus musculus OX=10090 GN=Hnrnpd PE=1 SV=2	1479	407	795	840	775	833	737	782	676	787	700	929	733	646	639	461	477	560	599	617	592	692	720	768
WT	NOX4	15798	60S ribosomal protein L27 OS=Mus musculus OX=10090 GN=Rpl27 PE=1 SV=2	1317	1240	1350	1188	1184	1260	1396	1298	1228	1310	1793	1034	1068	998	1013	794	654	678	1048	747	896	1260	1206	1264
WT	NOX4	17195	Ubiquitin-conjugating enzyme E2 N OS=Mus musculus OX=10090 GN=Ube2n PE=1 SV=1	9565	10017	7332	3826	3652	3631	3315	3395	3575	4788	5586	6849	3689	3618	3895	3385	3445	3766	5104	5683	3656	3050	3039	3413
WT	NOX4	13908	Histone H2B type 3-B OS=Mus musculus OX=10090 GN=Hist3h2bb PE=1 SV=1	2141	2084	1468	1332	1155	884	1486	1313	1204	1195	2954	2394	967	740	932	846	890	790	1619	1077	824	919	1346	1180
WT	NOX4	121064	Transcription elongation factor SPT5 OS=Mus musculus OX=10090 GN=Supt5h PE=1 SV=1	1647	3050	3370	1098	1022	1080	923	956	1003	1704	3844	1544	898	915	968	717	745	824	1041	1338	1226	1132	996	1233
WT	NOX4	29988	N(G)_N(G)-dimethylarginine dimethylaminohydrolase 2 OS=Mus musculus OX=10090 GN=Ddah2 PE=1 SV=1	3109	2972	2979	1001	854	961	1131	898	915	1390	1640	1526	795	771	708	551	584	551	1115	1113	1018	830	811	878
WT	NOX4	174237	Structure-specific endonuclease subunit SLX4 OS=Mus musculus OX=10090 GN=Slx4 PE=1 SV=1	935	729	725	358	246	203	336	254	179	619	791	1005	272	172	183	147	141	55	431	531	469	119	174	139
WT	NOX4	188630	MKIAA0533 protein (Fragment) OS=Mus musculus OX=10090 GN=mKIAA0533 PE=4 SV=2	472	56	575	244	127	93	181	223	166	123	308	444	73	59	100	3	8	1	179	302	243	90	87	78
WT	NOX4	187388	Phosphatidylinositol 3_4_5-trisphosphate-dependent Rac exchanger 1 protein OS=Mus musculus OX=10090 GN=Prex1 PE=1 SV=2	311	127	166	15	1	0	1	237	1	10	7	5	2	2	2	4	3	5	2	2	0	2	3	1
WT	NOX4	15229	Uncharacterized protein (Fragment) OS=Mus musculus OX=10090 GN=Sptan1 PE=2 SV=1	10319	8182	7837	5190	4707	5043	5713	5683	5353	6737	8728	7204	6246	6039	5567	5479	4926	4857	6062	5799	5795	5050	5145	5411
WT	NOX4	35334	Leucine-rich repeat-containing protein 59 OS=Mus musculus OX=10090 GN=Lrrc59 PE=1 SV=1	3583	3916	3696	4139	4258	4438	4375	4768	4709	3753	4503	4884	3815	4012	4046	2987	2779	2907	1736	3161	2226	4588	4595	4813
WT	NOX4	20481	60S ribosomal protein L11 OS=Mus musculus OX=10090 GN=Rpl11 PE=1 SV=4	3307	3280	3187	2358	2349	2361	2807	2642	3042	3488	4014	3963	2517	2157	2481	1648	1674	1513	3458	3491	3142	2627	2474	2634
WT	NOX4	19781	Actin-related protein 2/3 complex subunit 4 OS=Mus musculus OX=10090 GN=Arpc4 PE=1 SV=3	5799	6302	6294	3460	3043	3184	2709	2820	3106	3526	4031	4163	2690	2947	2923	3170	3353	3511	3473	3819	3548	2974	2879	2925
WT	NOX4	16860	NADH dehydrogenase [ubiquinone] 1 alpha subcomplex subunit 13 OS=Mus musculus OX=10090 GN=Ndufa13 PE=1 SV=3	3889	4164	4041	4604	4709	4585	3610	4044	3852	3598	4763	5089	3635	3607	3770	2992	3032	3084	2691	2637	2632	3732	4156	4193
WT	NOX4	47422	26S proteasome regulatory subunit 6B OS=Mus musculus OX=10090 GN=Psmc4 PE=1 SV=2	1071	606	749	374	391	425	514	472	419	678	1149	1211	464	429	373	502	464	335	680	232	749	452	328	426
WT	NOX4	11720	Cytochrome c_ somatic OS=Mus musculus OX=10090 GN=Cycs PE=1 SV=2	2478	3182	3195	3800	4042	4085	2276	2510	2417	2397	3443	3392	2122	2010	1959	2286	2265	2228	1603	1571	1529	1803	1745	1715
WT	NOX4	15480	Fatty acid-binding protein 5 OS=Mus musculus OX=10090 GN=Fabp5 PE=1 SV=3	3076	3550	3282	2260	1927	1937	1169	829	831	1373	1768	1936	979	852	758	1245	1249	924	1262	1331	1425	1041	1060	1048
WT	NOX4	73050	Choline O-acetyltransferase OS=Mus musculus OX=10090 GN=Chat PE=2 SV=2	599	590	604	164	145	121	365	257	150	578	550	516	319	296	215	220	220	255	177	316	187	192	78	47
WT	NOX4	97832	Calcium-transporting ATPase OS=Mus musculus OX=10090 GN=Atp2c1 PE=1 SV=1	154	137	190	130	92	140	191	164	141	3116	3688	4371	305	268	219	4	36	31	133	145	105	57	7	12
WT	NOX4	112473	Ephrin type-A receptor 8 OS=Mus musculus OX=10090 GN=Epha8 PE=1 SV=2	63	31	28	2213	2362	2279	1147	200	61	32	31	588	61	58	43	205	60	63	30	76	120	30	65	36
WT	NOX4	62087	Pyruvate kinase OS=Mus musculus OX=10090 GN=Pklr PE=2 SV=1	770	987	681	871	964	920	622	530	598	725	1086	741	573	529	524	585	621	690	610	1013	780	553	509	503
WT	NOX4	36393	Eukaryotic translation initiation factor 2 subunit 1 OS=Mus musculus OX=10090 GN=Eif2s1 PE=1 SV=3	864	1008	793	943	1031	1003	832	786	744	653	828	833	541	520	528	1041	1139	1181	430	405	363	668	638	657
WT	NOX4	219558	Endoribonuclease Dicer OS=Mus musculus OX=10090 GN=Dicer1 PE=1 SV=3	4820	4559	4301	2175	1803	1892	2474	2259	2608	3597	3392	3453	2552	2402	2521	1999	1965	1795	3180	3412	3101	1481	1387	1517
WT	NOX4	11425	ATP synthase subunit g_ mitochondrial OS=Mus musculus OX=10090 GN=Atp5mg PE=1 SV=3	18652	18723	18852	11252	11854	10909	10665	11924	11893	12666	16074	18425	11572	11306	11809	7364	7875	8323	7704	8057	8488	10952	10970	10724
WT	NOX4	101683	Mdm2-binding protein OS=Mus musculus OX=10090 GN=Mtbp PE=1 SV=1	1423	300	133	254	103	80	310	119	111	358	277	272	168	106	68	187	90	99	265	101	63	113	136	76
WT	NOX4	39906	Core histone macro-H2A.1 OS=Mus musculus OX=10090 GN=H2afy PE=1 SV=3	4211	3112	3281	3180	3828	4221	3889	4312	4384	4705	5858	6236	3466	3346	3135	2379	2358	2171	4956	3910	4444	3980	3762	3865
WT	NOX4	139481	Nck-associated protein 5-like OS=Mus musculus OX=10090 GN=Nckap5l PE=1 SV=1	7259	6808	7125	5069	4463	4612	4581	5039	4855	5490	6008	7595	4863	4890	4681	3880	3851	4008	4737	4603	4981	4014	4257	4246
WT	NOX4	19558	Serine/arginine-rich splicing factor 3 OS=Mus musculus OX=10090 GN=Srsf3 PE=1 SV=1	8650	10251	11292	6432	5956	6157	6612	6331	6254	8247	10115	10320	6429	6335	6760	5515	5603	5245	7174	7347	7342	4227	4010	4176
WT	NOX4	32536	Enoyl-CoA delta isomerase 1_ mitochondrial OS=Mus musculus OX=10090 GN=Eci1 PE=1 SV=2	1774	1732	1269	2164	2212	2101	1627	1627	1385	1980	2270	2367	1137	1087	915	1109	1101	831	1734	1859	1251	1520	1132	979
WT	NOX4	8236	ATP synthase subunit e_ mitochondrial OS=Mus musculus OX=10090 GN=Atp5me PE=1 SV=2	13195	13010	13116	4296	4258	4076	5097	4530	4529	7036	7718	10495	5258	5078	4692	5231	5111	4810	5840	6025	6392	3502	3383	3682
WT	NOX4	75395	Acetyl-coenzyme A synthetase OS=Mus musculus OX=10090 GN=Acss1 PE=2 SV=1	242	169	146	296	295	251	230	352	276	428	261	153	243	206	205	73	86	172	157	103	128	208	167	181
WT	NOX4	131539	TBC1 domain family member 8 OS=Mus musculus OX=10090 GN=Tbc1d8 PE=1 SV=2	598	346	221	1235	481	284	808	209	117	333	268	464	601	121	62	1142	322	38	67	162	187	116	66	88
WT	NOX4	39334	Calcium-binding protein 39-like OS=Mus musculus OX=10090 GN=Cab39l PE=1 SV=3	343	473	481	127	303	388	295	460	387	382	739	611	311	230	252	205	219	230	263	170	99	201	212	124
WT	NOX4	19576	Translationally-controlled tumor protein OS=Mus musculus OX=10090 GN=Tpt1 PE=1 SV=1	0	0	29	302	259	11	81	794	0	[illegible]	0	1419	131	113	113	142	121	93	76	63	174	155	149	106
WT	NOX4	34422	Small glutamine-rich tetratricopeptide repeat (TPR)-containing_ alpha_ isoform CRA_b OS=Mus musculus OX=10090 GN=Sgta PE=2 SV=1	525	213	336	58	68	62	45	47	109	16	331	348	26	18	35	45	49	103	9	194	7	55	69	54
WT	NOX4	9768	Cytochrome b-c1 complex subunit 8 OS=Mus musculus OX=10090 GN=Uqcrq PE=1 SV=3	7859	7743	8726	4075	4196	4368	3907	3913	4431	5465	5453	5987	3639	3756	3946	4778	4973	5171	4744	4707	4292	3901	4074	4711
WT	NOX4	19567	Ubiquitin-conjugating enzyme E2 variant 1 OS=Mus musculus OX=10090 GN=Ube2v1 PE=4 SV=1	7051	6222	5579	3498	2899	3353	3619	3183	3509	4646	4273	4921	3809	3421	3600	4209	4164	4216	3286	3697	3127	2890	2738	2771
WT	NOX4	34342	Thioredoxin-related transmembrane protein 2 OS=Mus musculus OX=10090 GN=Tmx2 PE=1 SV=1	1205	3037	2410	1764	2446	2251	2136	2543	2278	2108	2726	3081	1806	1796	2025	1332	1422	1536	1785	1810	1517	2273	2243	2352
WT	NOX4	102190	Kinesin-like protein KIF18A OS=Mus musculus OX=10090 GN=Kif18a PE=1 SV=1	1292	797	819	983	1021	1004	766	717	866	901	790	1090	733	707	764	766	670	555	923	549	543	783	723	813
WT	NOX4	152070	Capping protein_ Arp2/3 and myosin-I linker protein 3 OS=Mus musculus OX=10090 GN=Carmil3 PE=1 SV=2	532	700	659	354	257	220	299	370	336	301	305	451	291	346	331	212	210	321	283	209	309	293	335	422
WT	NOX4	115453	Protein phosphatase 1 regulatory subunit 12A OS=Mus musculus OX=10090 GN=Ppp1r12a PE=1 SV=2	1313	1541	1349	817	825	933	836	810	814	1403	1735	1794	728	708	712	721	599	681	1038	971	979	947	879	955
WT	NOX4	81695	Inhibitor of nuclear factor kappa-B kinase subunit epsilon OS=Mus musculus OX=10090 GN=Ikbke PE=1 SV=2	2442	3085	3483	1757	1468	1530	1377	1365	1226	2031	2687	4119	1411	1353	1240	714	678	779	2994	2742	2514	973	950	946
WT	NOX4	67776	Polyadenylate-binding protein OS=Mus musculus OX=10090 GN=Pabpc1l PE=2 SV=1	677	1252	2091	563	593	621	573	403	479	346	235	292	300	313	245	113	134	60	143	474	348	515	675	802
WT	NOX4	23480	Uncharacterized protein OS=Mus musculus OX=10090 GN=Gm5766 PE=2 SV=1	68	25	28	255	228	201	111	49	78	70	65	90	60	57	51	49	44	38	44	42	46	91	58	55
WT	NOX4	89502	Fibroblast growth factor receptor OS=Mus musculus OX=10090 GN=Fgfr4 PE=3 SV=1	0	0	0	19447	59519	14716	1373	0	265	0	441	200	1715	4668	4457	725	1725	1285	1249	2562	1456	1761	4628	2084
WT	NOX4	13417	NADH dehydrogenase [ubiquinone] 1 alpha subcomplex subunit 5 OS=Mus musculus OX=10090 GN=Ndufa5 PE=1 SV=3	3331	3621	2184	2314	2170	2127	2070	2092	1912	2332	2764	1933	2208	2062	2140	1732	1730	2066	1730	1919	2132	2090	1923	1959
WT	NOX4	122843	Constitutive coactivator of PPAR-gamma-like protein 1 OS=Mus musculus OX=10090 GN=FAM120A PE=1 SV=2	1690	1492	1378	1264	1261	1314	1049	1043	1220	916	1096	1116	772	813	844	899	1006	811	987	1042	1087	1227	1099	1150
WT	NOX4	52683	Cysteine protease ATG4C OS=Mus musculus OX=10090 GN=Atg4c PE=1 SV=2	1546	1141	1472	1569	1065	1244	988	972	895	738	864	1405	578	757	752	658	818	907	928	1052	1167	893	1219	1141
WT	NOX4	9291	Cytochrome c oxidase subunit 7A2_ mitochondrial OS=Mus musculus OX=10090 GN=Cox7a2 PE=1 SV=2	15800	17721	13563	6602	6706	6353	7375	9014	8109	10737	14842	12883	9920	10606	10331	5987	6018	6471	7413	8879	7278	7463	7532	7799
WT	NOX4	25796	Family with sequence similarity 213_ member A OS=Mus musculus OX=10090 GN=Fam213a PE=1 SV=1	1472	1360	1199	2257	2387	2475	1671	1771	1785	1465	1710	1866	1541	1461	1593	751	669	744	918	998	857	1909	1953	1890
WT	NOX4	71890	Tyrosine-protein kinase SYK OS=Mus musculus OX=10090 GN=Syk PE=1 SV=2	409	476	471	1070	361	583	675	796	380	751	1215	327	623	560	342	324	296	215	782	155	570	502	163	113
WT	NOX4	71646	Echinoderm microtubule-associated protein-like 2 OS=Mus musculus OX=10090 GN=Eml2 PE=1 SV=1	3424	2710	3026	1890	1848	1565	2737	2731	2786	4334	4654	5084	2577	2569	2139	653	566	560	1665	1810	1794	2250	2736	2940
WT	NOX4	12633	NADH dehydrogenase [ubiquinone] 1 alpha subcomplex subunit 7 OS=Mus musculus OX=10090 GN=Ndufa7 PE=1 SV=3	104	801	483	177	155	187	90	50	68	56	144	218	51	53	30	50	74	69	0	0	8	95	125	124
WT	NOX4	26041	Proteasome subunit alpha type-2 OS=Mus musculus OX=10090 GN=Psma2 PE=1 SV=3	948	1120	876	184	147	162	115	2337	141	155	85	36	165	133	157	46	49	47	98	148	247	133	122	104
WT	NOX4	27954	Uncharacterized protein OS=Mus musculus OX=10090 GN=Dhrs4 PE=2 SV=1	620	771	284	84	397	746	176	179	163	430	387	832	127	147	110	17	28	35	118	188	56	67	56	104
WT	NOX4	200284	Transcription elongation factor SPT6 OS=Mus musculus OX=10090 GN=Supt6h PE=1 SV=2	1388	2992	1825	161	12	69	23	15	48	14	1789	2486	11	7	22	29	19	7	242	166	79	18	6	22
WT	NOX4	46778	Actin-related protein 10 OS=Mus musculus OX=10090 GN=Actr10 PE=1 SV=1	2376	3520	3590	3416	3171	3671	3013	2467	2424	2664	2898	2588	2396	1940	1884	2841	3149	2816	2823	2454	2339	2455	2207	2251
WT	NOX4	23216	Rho-related GTP-binding protein RhoQ OS=Mus musculus OX=10090 GN=Rhoq PE=1 SV=2	892	704	398	640	806	751	654	745	765	715	912	1276	657	618	659	553	575	570	646	601	389	679	757	770
WT	NOX4	55379	Transmembrane protein 102 OS=Mus musculus OX=10090 GN=Tmem102 PE=1 SV=1	5039	5396	5300	5544	5576	5548	4675	5113	4998	5428	6244	7002	4507	4840	4862	3715	3780	3866	4033	4452	4610	4454	4621	4880
WT	NOX4	58902	Serine--tRNA ligase_ cytoplasmic OS=Mus musculus OX=10090 GN=Sars PE=1 SV=3	1592	1525	1525	1228	963	805	1052	970	1005	939	985	1122	832	749	688	991	906	997	989	953	982	805	812	837
WT	NOX4	167742	Peroxidasin homolog (Drosophila) OS=Mus musculus OX=10090 GN=Pxdn PE=2 SV=1	8813	7916	7955	3240	3126	3225	4569	3571	4003	5427	5094	4648	3792	3561	3786	3373	2895	3305	3578	3627	3459	2946	2728	3988
WT	NOX4	66379	Uncharacterized protein OS=Mus musculus OX=10090 GN=Farsb PE=2 SV=1	439	436	401	161	127	111	124	120	148	265	334	197	118	185	182	153	146	111	114	111	85	96	85	107
WT	NOX4	34406	Isoaspartyl peptidase/L-asparaginase OS=Mus musculus OX=10090 GN=Asrgl1 PE=1 SV=1	200	133	956	1057	925	769	509	361	378	433	1411	1193	446	342	328	261	245	117	271	642	116	507	436	448
WT	NOX4	55730	WD repeat-containing protein 37 OS=Mus musculus OX=10090 GN=Wdr37 PE=1 SV=1	1289	1274	502	586	388	436	98	20	110	426	843	839	96	78	66	298	244	118	702	331	317	120	37	36
WT	NOX4	124767	Phospholipase D1 OS=Mus musculus OX=10090 GN=Pld1 PE=1 SV=1	16	519	521	243	297	107	18	13	23	47	610	632	28	20	9	23	10	1	213	258	296	38	15	27
WT	NOX4	45418	Coiled-coil domain-containing protein 51 OS=Mus musculus OX=10090 GN=Ccdc51 PE=1 SV=1	85	115	49	10	14	13	15	17	0	19	41	0	26	23	0	0	0	0	0	0	0	35	0	0
WT	NOX4	17066	Mitochondrial fission 1 protein OS=Mus musculus OX=10090 GN=Fis1 PE=1 SV=1	2040	2278	2327	2160	2486	2284	1659	2101	1779	2327	2838	2858	1717	1895	1841	1216	1156	1222	2035	2010	2035	2053	2013	1998
WT	NOX4	214724	Tetratricopeptide repeat domain 6 OS=Mus musculus OX=10090 GN=Ttc6 PE=4 SV=1	4597	4129	4166	5233	6171	5423	6554	7239	7284	6940	7357	8581	4377	4621	4714	3237	3373	3195	3900	3844	3902	6906	6910	6946
WT	NOX4	65381	MKIAA0830 protein (Fragment) OS=Mus musculus OX=10090 GN=Endod1 PE=2 SV=1	964	1195	1071	1057	1002	1015	946	1016	875	1500	1685	1525	897	881	841	1034	998	840	449	310	197	1293	1168	1258
WT	NOX4	84495	DEAH (Asp-Glu-Ala-His) box polypeptide 16 OS=Mus musculus OX=10090 GN=Dhx16 PE=1 SV=1	10211	7199	10861	13454	12660	12899	13622	15088	15060	14847	16868	21812	10069	9938	9948	5398	5028	5096	10539	10364	11403	12279	11689	11520
WT	NOX4	50267	Septin-14 OS=Mus musculus OX=10090 GN=Sept14 PE=1 SV=3	1957	1837	1752	1346	1422	1446	2037	1609	1693	2786	3545	4835	1738	1375	1516	915	877	619	2843	2644	2467	874	764	973
WT	NOX4	40731	Phosphoserine aminotransferase OS=Mus musculus OX=10090 GN=Psat1 PE=2 SV=1	461	2374	2599	1357	1389	1402	1151	559	1760	1236	863	3126	844	550	1103	1063	1318	957	364	1277	1182	746	584	926
WT	NOX4	97910	Endothelial PAS domain-containing protein 1 OS=Mus musculus OX=10090 GN=Epas1 PE=1 SV=2	541	124	258	675	636	587	449	375	412	521	425	485	301	370	336	58	127	129	230	316	186	340	420	362
WT	NOX4	91812	Diacylglycerol kinase beta OS=Mus musculus OX=10090 GN=Dgkb PE=1 SV=2	0	0	0	135	137	220	141	82	117	0	0	0	0	0	13	0	0	0	0	0	0	4	0	0
WT	NOX4	112371	PHD finger protein 20-like protein 1 OS=Mus musculus OX=10090 GN=Phf20l1 PE=1 SV=1	1053	1437	1465	1151	1363	1345	1506	1701	1643	2012	2045	1869	1261	1654	1458	1177	1262	1239	1288	1330	1221	1013	1036	1066
WT	NOX4	147024	Regulator of G-protein signaling 22 OS=Mus musculus OX=10090 GN=Rgs22 PE=1 SV=1	294	501	414	405	436	495	346	369	336	634	514	599	442	378	295	352	290	259	471	331	268	316	339	276
WT	NOX4	28373	Transmembrane protein 33 OS=Mus musculus OX=10090 GN=Tmem33 PE=1 SV=1	1497	1859	1830	2235	2221	2279	1822	2183	2101	2343	2744	3067	1619	1768	1525	1691	1647	1772	657	570	525	2262	2303	2479
WT	NOX4	87617	AFG3-like protein 1 OS=Mus musculus OX=10090 GN=Afg3l1 PE=1 SV=2	3679	3813	3459	3338	2026	2008	1441	1679	2828	2739	3669	3331	1697	1915	2296	1251	1254	1505	3174	3335	2546	1783	1824	1601
WT	NOX4	10331	Barrier-to-autointegration factor OS=Mus musculus OX=10090 GN=Banf1 PE=1 SV=1	992	1275	1353	3379	1321	2077	1217	1332	1608	893	1679	1464	672	691	689	343	451	515	1586	1374	1604	1440	1466	2037
WT	NOX4	65331	Coiled-coil domain-containing protein 63 OS=Mus musculus OX=10090 GN=Ccdc63 PE=2 SV=2	166	118	156	454	368	374	415	361	368	370	310	350	288	272	256	127	100	35	19	273	233	216	240	209
WT	NOX4	84137	Uncharacterized protein OS=Mus musculus OX=10090 PE=2 SV=1	842	877	915	334	128	118	443	222	76	711	1040	660	287	137	73	600	358	133	278	354	300	164	107	16
WT	NOX4	145973	WD repeat domain 17 OS=Mus musculus OX=10090 GN=Wdr17 PE=1 SV=1	26	56	0	149	70	44	32	0	26	15	107	82	0	0	0	26	6	5	130	5	0	0	2	0
WT	NOX4	19911	Myosin regulatory light polypeptide 9 OS=Mus musculus OX=10090 GN=Myl9 PE=1 SV=3	4447	74	52	40	0	0	46	0	0	201	0	0	0	0	0	0	0	0	42	0	0	0	0	0
WT	NOX4	35626	ATP-dependent (S)-NAD(P)H-hydrate dehydratase OS=Mus musculus OX=10090 GN=Naxd PE=1 SV=1	2603	2777	2333	1745	1754	1855	1798	1648	1587	1983	1975	2605	1619	1532	1409	1490	1506	1429	1971	1809	1813	1738	1520	1627
WT	NOX4	20981	Lactoylglutathione lyase OS=Mus musculus OX=10090 GN=Glo1 PE=1 SV=1	982	1243	1135	1425	1452	1368	1185	1274	1171	1148	1320	1266	1046	1110	1057	1135	1108	1011	405	367	317	1148	1097	1127
WT	NOX4	57596	Coiled-coil domain-containing protein 61 OS=Mus musculus OX=10090 GN=Ccdc61 PE=1 SV=1	1200	1010	374	615	537	478	601	433	449	545	558	497	465	408	368	476	321	249	264	263	286	532	507	475
WT	NOX4	78528	Zinc finger protein 612 OS=Mus musculus OX=10090 GN=Zfp612 PE=4 SV=1	4357	2146	2334	1234	1051	972	1226	984	953	1905	2000	3221	1090	991	1019	1177	1050	1062	1766	1570	1621	967	844	967
WT	NOX4	221412	Nascent polypeptide-associated complex subunit alpha_ muscle-specific form OS=Mus musculus OX=10090 GN=Naca PE=1 SV=2	253	160	228	586	218	212	119	147	116	204	178	95	116	141	97	104	275	148	95	178	77	83	113	76
WT	NOX4	212217	MKIAA0530 protein (Fragment) OS=Mus musculus OX=10090 GN=Zfp292 PE=2 SV=1	7245	6062	9703	1743	1703	1598	2561	2259	1811	2884	3307	4684	2444	1902	2122	998	951	1021	2518	2543	2433	1861	1583	1826
WT	NOX4	34734	Uracil phosphoribosyltransferase homolog OS=Mus musculus OX=10090 GN=Uprt PE=1 SV=1	839	532	480	4738	5023	4893	5020	5207	5215	3175	3412	3742	3075	2664	2351	1376	1344	1201	159	114	127	2528	2503	2136
WT	NOX4	29513	Protein DEK (Fragment) OS=Mus musculus OX=10090 GN=Dek PE=1 SV=1	1400	1197	1171	317	194	106	242	197	180	104	261	394	186	63	68	85	37	55	345	359	140	286	231	321
WT	NOX4	45747	Interferon-activable protein 205-A OS=Mus musculus OX=10090 GN=Ifi205a PE=2 SV=1	4814	1054	5270	421	472	396	312	325	328	541	437	340	351	341	211	346	285	385	348	204	299	351	421	144
WT	NOX4	81642	Acetyl-coenzyme A synthetase_ cytoplasmic OS=Mus musculus OX=10090 GN=Acss2 PE=1 SV=1	1476	1051	1179	1010	918	857	769	595	820	869	709	1199	737	788	844	830	798	884	567	586	510	748	803	1008
WT	NOX4	105606	DNA repair protein complementing XP-C cells homolog OS=Mus musculus OX=10090 GN=Xpc PE=1 SV=2	2444	2340	2395	1876	2006	2074	1473	1570	1557	1626	1466	1493	1182	1275	1396	1391	1432	1688	1351	1347	1431	1506	1767	1825
WT	NOX4	33416	Exocyst complex component 7 (Fragment) OS=Mus musculus OX=10090 GN=Exoc7 PE=1 SV=1	783	697	650	661	796	821	653	817	788	755	823	716	598	631	738	306	441	328	564	638	526	695	727	678
WT	NOX4	11589	60S acidic ribosomal protein P1 OS=Mus musculus OX=10090 GN=Rplp1 PE=1 SV=1	5405	5604	4756	8237	7468	7547	7421	6269	6050	3777	5269	6574	5211	4757	4633	2938	3247	3316	3700	4634	4360	5623	6203	5190
WT	NOX4	58742	Calpain-10 OS=Mus musculus OX=10090 GN=Capn10 PE=3 SV=1	1875	3186	1868	5562	3802	3133	5439	4253	3542	3824	6552	6221	2951	2518	1788	4262	3590	2339	2687	2546	1982	3573	2571	1966
WT	NOX4	54290	Glutathione reductase_ mitochondrial OS=Mus musculus OX=10090 GN=Gsr PE=1 SV=3	3647	3081	2621	2177	1736	1470	1530	1221	1370	1621	2001	2565	1214	1221	1186	1294	1299	1585	1723	1775	1800	1124	965	1283
WT	NOX4	53286	Predicted gene_ EG210853 OS=Mus musculus OX=10090 GN=Zfp947 PE=2 SV=1	1176	432	750	1037	857	704	1108	672	946	912	474	690	673	491	577	412	227	197	229	54	193	510	601	895
WT	NOX4	16935	Ubiquitin-conjugating enzyme E2 L3 (Fragment) OS=Mus musculus OX=10090 GN=Ube2l3 PE=1 SV=1	144	462	405	847	350	428	346	205	343	78	95	150	178	178	168	163	128	149	36	71	15	329	303	259
WT	NOX4	87112	Protein PAT1 homolog 1 OS=Mus musculus OX=10090 GN=Patl1 PE=1 SV=2	16	2	17	47	57	50	95	33	40	67	56	100	60	12	27	9	4	4	38	3	22	46	15	14
WT	NOX4	94046	Axin-2 OS=Mus musculus OX=10090 GN=Axin2 PE=1 SV=2	613	528	357	21	75	102	70	28	43	175	342	437	97	20	24	32	18	28	353	370	222	24	11	13
WT	NOX4	24446	Maleylacetoacetate isomerase OS=Mus musculus OX=10090 GN=Gstz1 PE=1 SV=1	946	4715	7169	399	482	520	434	480	462	596	675	918	343	285	378	331	327	371	527	444	393	324	278	281
WT	NOX4	139666	Dermatan-sulfate epimerase-like protein OS=Mus musculus OX=10090 GN=Dsel PE=2 SV=1	592	827	515	3	0	55	49	34	56	327	189	217	27	19	6	0	0	0	6	250	94	23	3	8
WT	NOX4	120676	MCG130893 OS=Mus musculus OX=10090 GN=Zbtb11 PE=1 SV=1	305	813	945	232	260	256	135	147	175	87	112	70335	171	98	177	144	89	70	93	61	117	125	184	121
WT	NOX4	57701	Cytochrome P450_ family 2_ subfamily j_ polypeptide 13 OS=Mus musculus OX=10090 GN=Cyp2j13 PE=2 SV=1	0	474	1014	40	31	23	6	0	0	0	0	9	0	0	0	0	0	0	0	0	0	3	0	2
WT	NOX4	32579	Uncharacterized protein OS=Mus musculus OX=10090 GN=Txnl1 PE=2 SV=1	0	0	0	1253	789	751	9	2	0	0	0	0	171	181	151	24	24	3	566	675	438	11	4	4
WT	NOX4	96928	Armadillo repeat-containing protein 3 OS=Mus musculus OX=10090 GN=Armc3 PE=1 SV=1	104	16	0	342	491	457	299	445	400	252	71	206	201	229	321	0	0	6	0	36	11	45	38	60
WT	NOX4	15420	28S ribosomal protein S16_ mitochondrial OS=Mus musculus OX=10090 GN=Mrps16 PE=1 SV=1	17638	24009	21576	1053	748	757	694	1205	718	1957	4494	1485	735	1315	840	726	692	893	660	2131	1632	1080	860	899
