# Supplementary material for: NOX4-derived ROS are neuroprotective by balancing intracellular calcium stores
Source: Cell Mol Life Sci. 2023 Apr 21;80(5):127. doi: 10.1007/s00018-023-04758-z (PMC10119225; doi:10.1007/s00018-023-04758-z)
Supplement: Supplementary file 2 — Supplementary file2 (PDF 368 KB) [file 18_2023_4758_MOESM2_ESM.pdf]

NOX4-derived ROS are neuroprotective by balancing intracellular calcium stores

Lukas Gola[1,], Laura Bierhansl[1,], Júlia Csatári[1], Christina B. Schroeter[2], Lisanne Korn[1], Venu Narayanan[1], Manuela Cerina[1], Sara Abdolahi[3], Anna Speicher[1], Alexander M. Hermann[2], Simone König[4], Albena T. Dinkova-Kostova[5], Tawfeeq Shekh-Ahmad[6], Sven G. Meuth[2], Heinz Wiendl[1], Ali Gorji[3,7], Matthias Pawlowski[1], Stjepana Kovac[1, §]

[1] Department of Neurology with Institute of Translational Neurology, University Hospital Münster, 48149 Münster, Germany.

[2] Department of Neurology, Medical Faculty, Heinrich Heine University Düsseldorf, Moorenstraße 5, 40225 Düsseldorf, Germany.

[3] Shefa Neuroscience Research Center, Khatam Alanbia Hospital, Tehran, Iran.

[4] Core Unit Proteomics, Interdisciplinary Center for Clinical Research, Medical Faculty, University of Münster, 48149 Münster, Germany.

[5] Division of Cellular Medicine, School of Medicine, University of Dundee, Dundee DD1 9SY, UK.

[6] Institute for Drug Research, The School of Pharmacy, Faculty of Medicine, The Hebrew University of Jerusalem, Jerusalem 91120, Israel.

[7] Epilepsy Research Center, Westfälische Wilhelms-Universität Münster, 48149 Münster, Germany.

§ Corresponding author, Stjepana.Kovac@ukmuenster.de

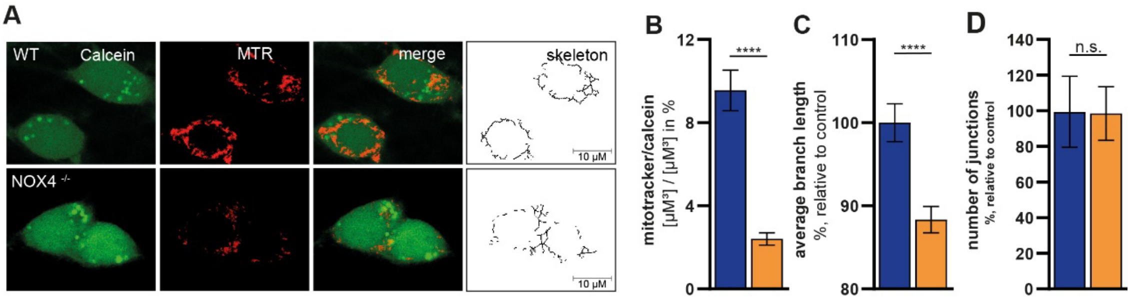

Figure S1. Mitochondrial volume

a Representative Images of glio-neuronal cells stained with Calcein and MTR**. b** Mitochondrial volume expressed as percentage of cell volume (NOX4$^{-/-}$, n= 17; WT, n= 15). **c-d** Further analysis of mitochondrial network of glio-neuronal cells.

Data information: Data are mean ± SEM; Student's t-test; n ≙ number of glio-neuronal cells out of 3 independent neuronal preparations, each with 2 CS; ****p<0.0001.
